# Targeting serine synthesis pathway to reverse paclitaxel resistance in NSCLC with combination of paclitaxel and anlotinib

**DOI:** 10.1186/s13046-025-03627-w

**Published:** 2026-01-05

**Authors:** Mengting Yu, Yanyun Hong, Qingshan Pan, Pengwu Zheng, Yingxing He, Wufu Zhu, Shan Xu, Qiaoli Lv

**Affiliations:** 1 Jiangxi Provincial Key Laboratory of Drug Design and Evaluation, School of Pharmacy, Jiangxi Science & Technology Normal University, Nanchang, Jiangxi 330013 China; 2 Jiangxi Key Laboratory of Oncology (2024SSY06041), JXHC Key Laboratory of Tumour Metastasis, JXHC Key Laboratory of Thoracic Oncology. Jiangxi Cancer Hospital & Institute, the Second Affiliated Hospital of Nanchang Medical College, Nanchang, Jiangxi 330029 China

**Keywords:** Non-small cell lung cancer, Serine synthesis pathway, Paclitaxel resistance, Anlotinib

## Abstract

**Background:**

Paclitaxel (PTX) serves as a first-line chemotherapeutic agent for the treatment of advanced non-small cell lung cancer (NSCLC). However, the emergence of drug resistance poses a significant threat to patient survival. The serine synthetic pathway (SSP) has been implicated in drug resistance across various cancers and is notably activated in NSCLC. Nevertheless, its role in PTX resistance remains poorly understood.

**Methods:**

In this study, we investigated the influence of the SSP on PTX resistance in NSCLC and explored a novel combination therapeutic strategy involving PTX and anlotinib to reverse NSCLC drug resistance. Specifically, using integrated transcriptomic and metabolomic analyses along with *in vitro* and *in vivo* experimental approaches, we aimed to elucidate the regulatory role of activated SSP in PTX resistance and to determine whether the combination of anlotinib and PTX can overcome PTX resistance in NSCLC through modulation of the SSP.

**Results:**

We found that SSP activation drives PTX resistance by promoting the proliferation of PTX-resistant NSCLC cells, increasing the expression and transport function of P-glycoprotein (P-gp), inducing epithelial-to-mesenchymal transition (EMT), and maintaining redox homeostasis. Anlotinib synergizes with PTX by suppressing SSP. This leads to attenuated glycolysis, disruption of the AKT/ERK proliferative signaling pathway, inhibition of P-gp expression and function, reversal of EMT, and redox imbalance, which subsequently elevates reactive oxygen species (ROS) levels and activates the mitochondrial apoptosis pathway, ultimately inducing apoptosis.

**Conclusion:**

Collectively, our study demonstrates that anlotinib combined with PTX, via SSP inhibition, is a promising strategy for overcoming PTX resistance in NSCLC.

**Graphical abstract:**

**Figure:**
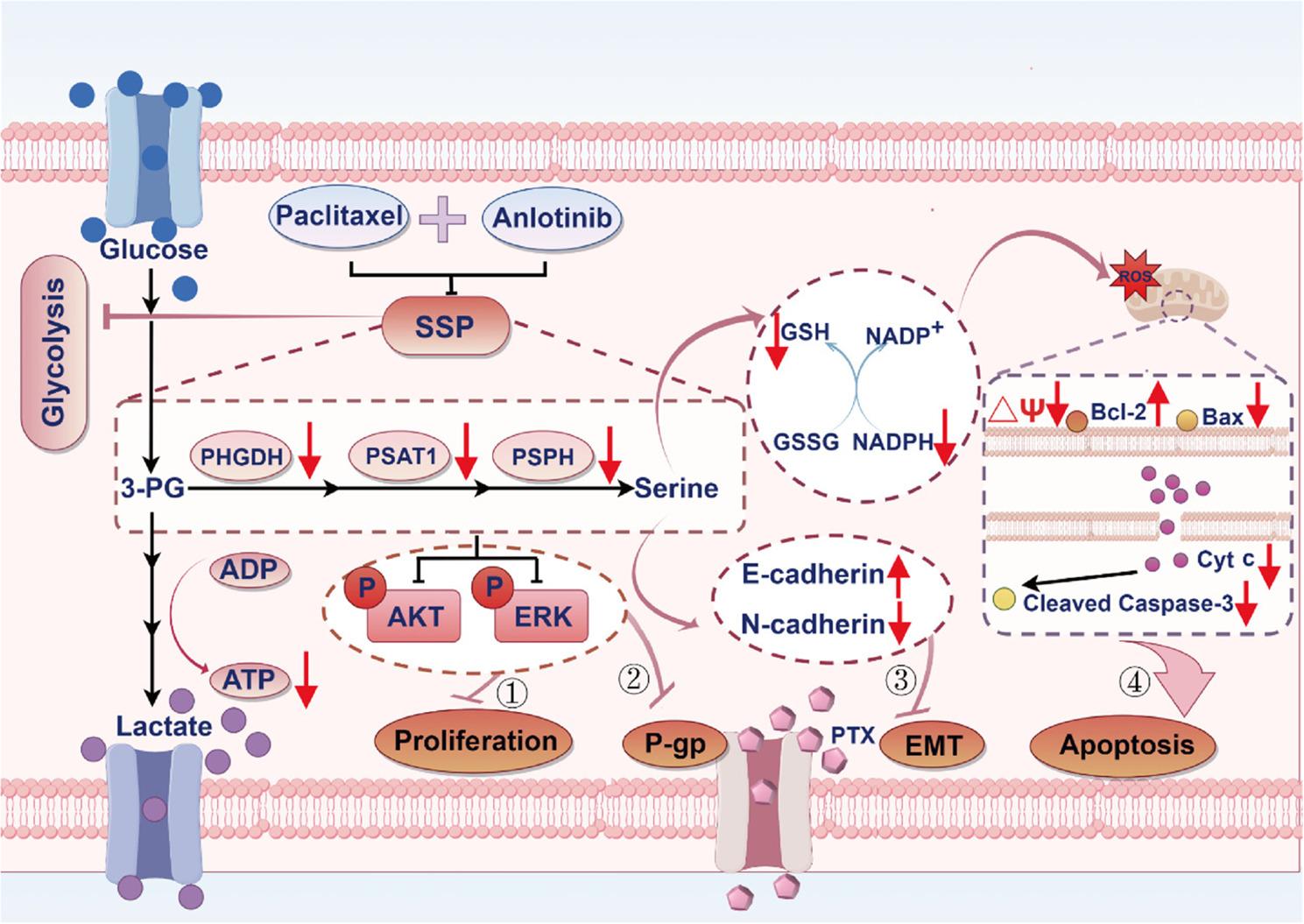
The combination of anlotinib and PTX effectively suppresses the SSP in A549/PTX cells. This suppression results in the following effects: (1) disruption of AKT/ERK proliferation signaling pathway transmission; (2) inhibition of P-gp expression and its efflux function; (3) blockade of the EMT process; (4) activation of the mitochondrial apoptosis pathway, thereby inducing cell apoptosis. Furthermore, the inhibition of SSP also exerts a certain degree of suppression on the glycolytic activity of A549/PTX cells.

**Supplementary Information:**

The online version contains supplementary material available at 10.1186/s13046-025-03627-w.

## Introduction

Non-small cell lung cancer (NSCLC) represents one of the most prevalent malignant tumors globally. Around 85% of patients are diagnosed at an advanced stage, resulting in a 5-year survival rate of less than 15% [1]. Currently, chemotherapy remains the primary treatment modality for NSCLC. Paclitaxel (PTX) has been extensively utilized as a standard first-line chemotherapy agent due to its notable anti-tumor efficacy [2]. However, the development of PTX resistance is a prevalent challenge in the chemotherapy of NSCLC patients, posing a significant threat to treatment outcomes and adversely impacting patient health and quality of life [3]. PTX resistance is a multifaceted process characterized by the activation of the AKT/ERK signaling pathway [4], overexpression of P-glycoprotein (P-gp) [5], dysregulation of epithelial-mesenchymal transition (EMT) [6], evasion of apoptosis [7], and metabolic disturbances [8]. These mechanisms interact with one another and ultimately contribute to the failure of chemotherapy in NSCLC. Therefore, it is imperative to identify novel therapeutic targets to combat PTX resistance in NSCLC and to develop innovative strategies to enhance chemotherapy sensitivity.

Reprogramming of serine metabolism has been implicated in all stages of tumorigenesis. Abnormal serine metabolism, particularly the enhancement of the serine synthesis pathway (SSP), is commonly observed in lung cancer [9]. The SSP serves as a crucial turning point in the glycolytic pathway, converting the glycolytic intermediate 3-phosphoglycerate (3-PG) into serine via three critical enzymes: phosphoglycerate dehydrogenase (PHGDH), phosphoserine aminotransferase 1 (PSAT1), and phosphoserine phosphatase (PSPH) [10]. Research indicates that cancer cells frequently activate SSP to satisfy their demand for rapid growth [11]. However, excessive activation of SSP may lead to tumor malignancy, driving uncontrolled proliferation, metastasis, immunosuppression, and drug resistance [12]. Furthermore, SSP plays a vital role in maintaining redox homeostasis by regulating glutathione (GSH) production and nicotinamide adenine dinucleotide phosphate (NADPH) regeneration; activated SSP aids cancer cells in protecting against oxidative stress damage [13]. Inhibition of the SSP pathway can disrupt intracellular redox homeostasis, leading to reactive oxygen species (ROS) generation. Elevated ROS levels may activate apoptosis-related signaling pathways in cancer cells, ultimately inducing tumor cell apoptosis [14]. Given these findings, SSP inhibition emerges as a promising strategy to overcome PTX resistance in NSCLC.

Currently, combination therapies represent one of the most effective and safest options for cancer treatment, as they are less likely to lead to drug resistance and demonstrate greater efficacy compared to single-agent regimens [4]. Anlotinib is a novel oral multi-target receptor tyrosine kinase inhibitor. Its distinctive molecular structure allows it to significantly inhibit the phosphorylation of kinases such as VEGFR, FGFR, and PDGFR, thereby obstructing the downstream AKT/ERK signaling pathway and markedly inhibiting the proliferation, migration, and tumor growth of cancer cells *in vivo* [15, 16]. Approved as third-line NSCLC therapy in China, anlotinib shows potent anti-tumor activity and tolerable safety in clinical trials [17]. Compared to anlotinib monotherapy, the combination of anlotinib with crizotinib [18], gefitinib [19], osimertinib [20], and other chemotherapeutic agents can yield superior anticancer efficacy in NSCLC. However, the synergistic effect of anlotinib combined with PTX on PTX-resistant NSCLC cells and the specific mechanism of action remain unclear.

In this study, we demonstrate that activated SSP plays a crucial role in maintaining PTX resistance in NSCLC cells. Our findings indicate that the combination of anlotinib and PTX inhibits the AKT/ERK proliferative signaling pathway while activating the mitochondrial apoptosis pathway through suppression of SSP in PTX-resistant NSCLC cells. Furthermore, the inhibition of the SSP decreases P-gp expression and transport function, reverses the EMT process, and ultimately overcomes PTX resistance in NSCLC cells. These results suggest that combining PTX with anlotinib represents an effective strategy to overcome PTX resistance in NSCLC. Our work provides compelling evidence and a theoretical foundation for addressing PTX resistance in NSCLC.

## Materials and methods

### Materials

Paclitaxel (CAS No.33069-62-4), anlotinib (CAS No.1360460-82-7) and NCT-503 were (CAS No.1916571-90-8) purchased from InnoChem (Beijing, China). NADPH/NADP^+^ assay kit (Elabscience, E-BC-K803-M). ATP assay kit (Servicebio, G4309-48T). T-GSH/GSSG assay kit (Elabscience, E-BC-K097-M). ATP assay kit (Servicebio, G4309-48T). ROS assay kit (Biosharp, BL714A). Lactate assay kit (Solarbio, BC2235). Phosphoglycerate Dehydrogenase (PHGDH) activity assay kit (Abcam, ab273328). Antibodies were sourced from the following providers: PHGDH (Proteintech,14719-1-AP), AKT (Servicebio, GB15689), PSAT1 (Proteintech, 10501-1-AP), P-gp (Proteintech, 22336-1-AP), p-AKT (Servicebio,GB150002), PSPH (Proteintech, 14513-1-AP), ERK (Servicebio, GB12087), p-ERK (Servicebio, GB11004), Bax (Affinity, AF0120), E-cadherin (Servicebio, GB12038), N-cadherin (Servicebio, GB12135), Bcl-2 (Affinity, AF6139), Cytochrome c (Proteintech, 10993-1-AP), Cleaved Caspase-3 (Affinity, AF7022), GAPDH (Proteintech, 60004-1-Ig), β-Tubulin (Servicebio, GB152667), β-actin (Servicebio, GB-15003).

### ATP and lactate detection

The ATP assay kit (Servicebio, G4309-48T) and lactate assay kit (Solarbio, BC2235) were used to measure ATP and lactate levels. Cell lysates or tumor tissue homogenates were prepared, and supernatants were analyzed following the suppliers’ procedures.

### ROS detection

To evaluate the intracellular ROS in A549/PTX cells, a ROS assay kit from Biosharp (Beijing, China) was employed. The intensity of green fluorescence, indicating the amount of ROS present in the cells, was visualized by fluorescence microscope and quantified with ImageJ software. Furthermore, intracellular ROS concentrations were quantified using flow cytometry and the resulting data were processed with FlowJo software.

### Western blotting

After exposing cells to specified drug concentrations for 72 h, cell lysis was performed using RIPA buffer. For western blotting analysis, proteins were extracted as described previously [21].

### Transcriptome sequencing

A549 and A549/PTX cells were exposed to designated drugs and underwent transcriptome profiling via RNA sequencing (RNA-seq). Library construction and next-generation sequencing were performed by Wuhan Mavie Metabolic Biotechnology Co., Ltd. in Wuhan, China. For quality control, FastQC evaluated RNA-seq data, with further processing conducted on Metware Cloud (https://cloud.metware.cn), an online bioinformatics platform.

### Metabolomics

Serum from nude mice was collected, and metabolites were extracted with 20% acetonitrile in methanol. Data were collected via a UPLC-MS/MS system by Wuhan Mavie Metabolic Biotechnology Co., Ltd. (Wuhan, China). Using established metabolite standards, the MWDB (Metware Database) was constructed to facilitate qualitative and quantitative analysis of mass spectrometry data.

### Animal experiments

All animal procedures were approved by the Animal Ethics Committee of Jiangxi University of Science and Technology (Y202451). Male BALB/c-nu nude mice (5-week-old) were obtained from SPF Biotechnology Co., Ltd. (Beijing). A549/PTX cells (2 × 10⁷ cells/mL in PBS) were subcutaneously injected into the right axilla. Tumor volume was measured every two days using the formula: 0.5 × length × width². When tumors reached ~ 100 mm³, mice were randomized into five groups: (1) Vehicle control; (2) Paclitaxel (10 mg/kg, i.p.); (3) Anlotinib (6 mg/kg, p.o.); (4) Paclitaxel + Anlotinib; (5) NCT-503 (30 mg/kg, i.p.).Treatments were administered every other day for two weeks. After treatment, mice were euthanized, and tumor tissues with major organs were collected for H&E staining and immunohistochemistry by Wuhan Xavier Biotechnology.

### Statistical analysis

The findings were derived from at least three independent trials, all data represent mean ± SD. The analysis was conducted using the GraphPad Prism 7.0 software. To gauge the statistical validity of our results, we utilized the Student’s t-test for comparisons between two groups and ANOVA for more complex multi-group comparisons. We considered a result statistically significant under the following thresholds: anything above 0.05 was deemed not significant (ns), while *p < 0.05, **p < 0.01, ***p < 0.001, and ****p < 0.0001 signified progressively higher levels of significance.

### Other methods

Detailed methods for cell culture, viability assays, colony-formation assays, EdU-incorporation assays, Rh123 accumulation, flow cytometry, wound-healing assays, transwell assays, and calcein-AM/PI staining, Enzyme activity assay are provided in the Supporting Information.

## Results

### Anlotinib and paclitaxel have synergistic therapeutic effect on PTX-resistant NSCLC cells

**Fig. 1:**
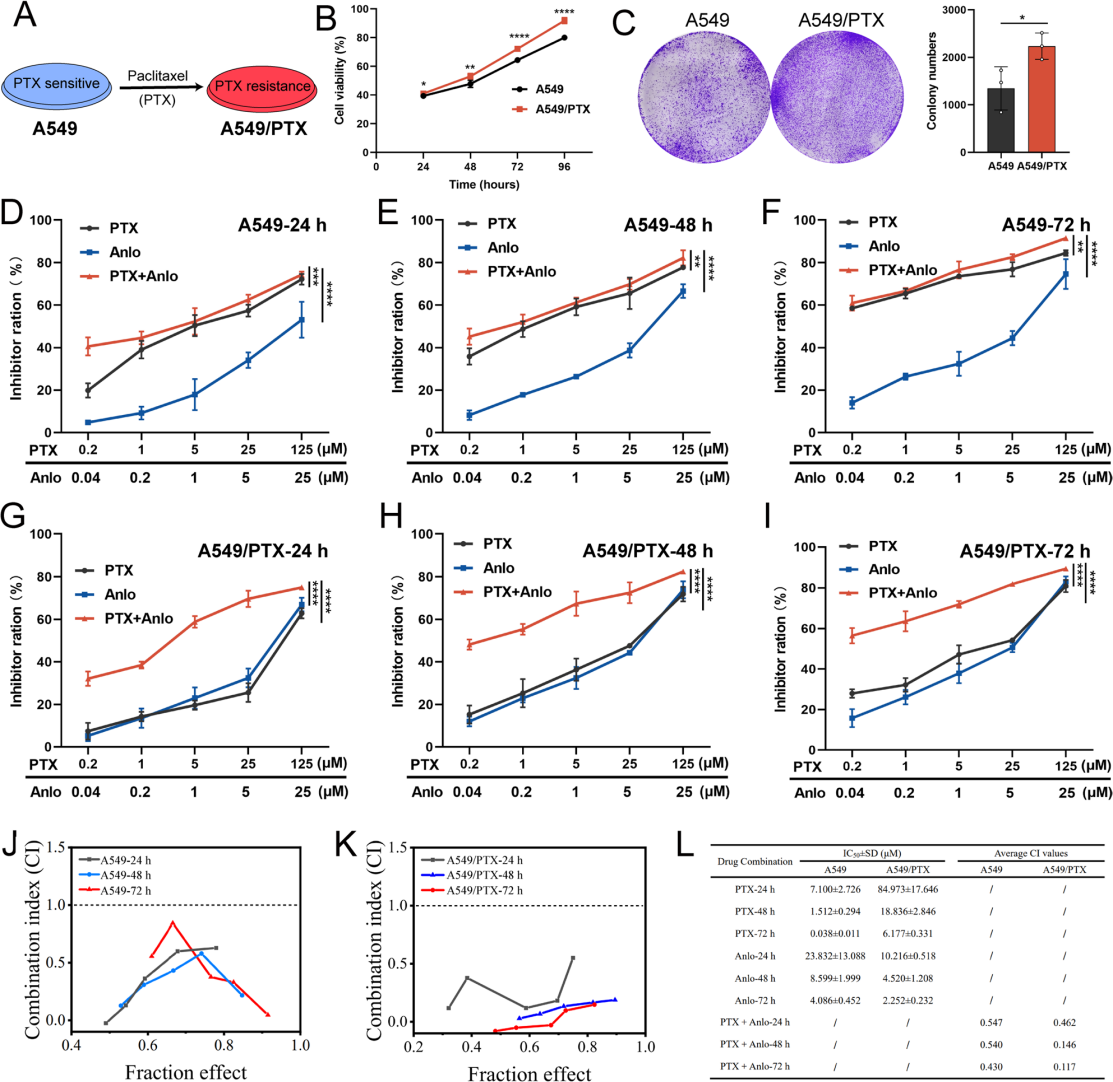
Growth-inhibitory effect of the combination of anlotinib and paclitaxel on PTX-sensitiveand PTX-resistant NSCLC cell lines. A Schematic representation of A549/PTX cell generation. B CCK-8 assay (n= 6) and Ccolony formation assay (n= 3) assessing proliferation of A549 andA549/PTX cells. D-F Growth inhibition of A549 cells (n= 3) treated with PTX and anlotinib assingle agents or in combination for 24-72 h. G-I Growth inhibition of A549/PTX cells (n= 3)treated with PTX and anlotinib as single agents or in combination for 24-72 h. J CI values of PTXplus anlotinib in A549 cells. K CI values of PTX plus anlotinib in A549/PTX cells.L Table of IC values for PTX, anlotinib, and their combination, and CI values following drug treatment inNSCLC cell lines

A549 cells developed resistance to PTX following prolonged exposure, resulting in the formation of A549/PTX cells (Fig. 1A) and subsequently, these cells showed a marked increase in proliferation over the parental line (Fig. 1B-C). In this research, we first evaluated the effects of PTX and anlotinib, both individually and in combination, on the growth activity of A549 and A549/PTX cells. For A549 cells, the combination treatment significantly reduced cell proliferation compared to single-agent treatments, with the effect being both concentration- and time-dependent (Fig. 1D-F). The same methodology was applied to A549/PTX cells, yielding trends consistent with those observed in A549 cells (Fig. 1G-I). The combination index (CI) was used to evaluate the drug interaction, revealing that the combination of anlotinib and PTX exhibited synergistic effects on both A549 cells (Fig. 1J) and A549/PTX cells (Fig. 1K). Notably, despite the high resistance of A549/PTX cells to PTX (with an IC_50_ of 6.177 μM, representing a 162-fold increase over the 0.038 μM IC_50_ in parental A549 cells), the combination of anlotinib and PTX nonetheless exhibited a stronger synergistic effect in these cells, as evidenced by a lower mean CI value (Fig. 1L).

### Increased PHGDH expression and activation of serine synthesis pathway is associated with paclitaxel resistance in NSCLC cells

**Fig. 2:**
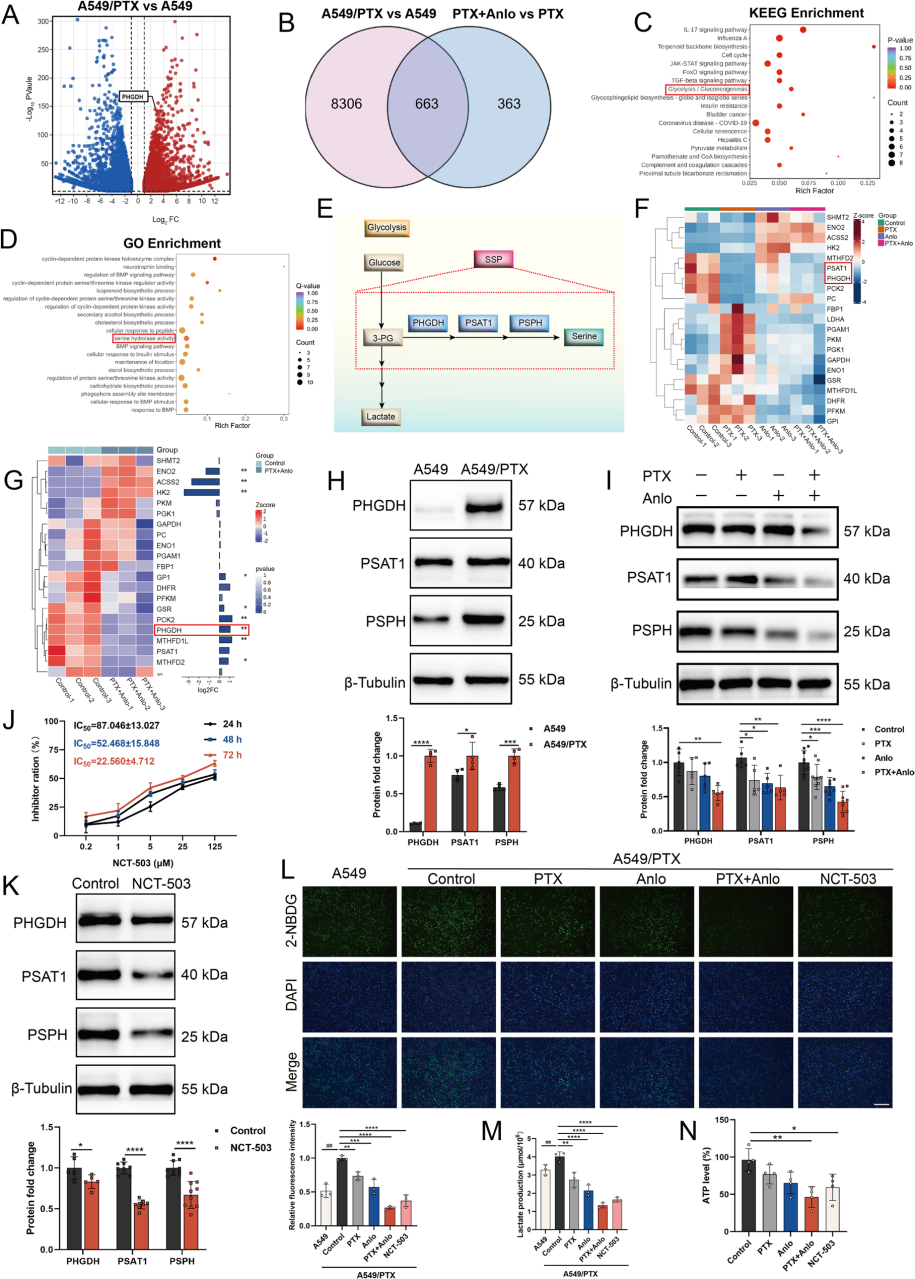
The combination of anlotinib and paclitaxel inhibits the SSP of A549/PTX cells. **A** The volcano plot for DEGs reveals that there is a significant upregulation of the PHGDH gene in A549/PTX cells when compared to A549 cells. **B** Venn diagram analysis reveals the overlap of DEGs. **C** KEGG enrichment analysis of DEGs. **D** GO enrichment analysis of DEGs. **E** A simplified schematic diagram illustrates the three key enzymes involved in SSP: PHGDH, PSAT1, and PSPH. **F** Cluster heatmap analysis of the key genes involved in glycolysis and the SSP across all samples. **G** Focused analysis of key genes in the Control and PTX+Anlo groups identified a significant downregulation of PHGDH. **H** The differences expression of key SSP proteins in A549 and A549/PTX cells were measured by western blotting. **I** Western blotting was used to assess the expression of key SSP proteins following treatment with PTX (6 μM), anlotinib (2 μM), or their combination in A549/PTX cells. **J** MTT assay evaluates the proliferation inhibitory activity of NCT-503 on A549/PTX cells (*n* = 4). **K** Western blotting was used to examine the differences expression of key SSP proteins after treatment with NCT-503 (22 μM) in A549/PTX cells. **L** The glucose uptake ability (scale bar: 500 μm; *n* = 3) and **M** the lactate content (*n* = 3) of A549 and A549/PTX cells were measured following treatment with PTX (6 μM), anlotinib (2 μM), their combination, or NCT-503 (22 μM). **N** ATP content detection in A549/PTX cells (*n* = 4)

In order to explore the potential factors contributing to the resistance observed in A549/PTX cells against PTX, we first conducted transcriptomic analysis aimed at identifying the variations in gene expression between A549 and A549/PTX cell lines. Notably, it was found that PHGDH was significantly upregulated in A549/PTX cells (Fig. 2A). Numerous previous studies have demonstrated that PHGDH is frequently overexpressed in a wide range of tumors and it has been established that high levels of PHGDH are closely linked to the processes of tumorigenesis, the development of drug resistance, and a poor prognosis for patients [22]. Moreover, elevated levels of PHGDH can promote tumor growth and inhibit apoptosis in cancer cells induced by chemotherapy drugs through the activation of the SSP [23].To further investigate the effect of anlotinib on the gene expression of A549/PTX cells following PTX treatment, we identified 363 differentially expressed genes (DEGs) using a Venn diagram (Fig. 2B). Subsequent Kyoto Encyclopedia of Genes and Genomes (KEGG) enrichment analysis revealed that the PTX-anlotinib combination impacted the glycolysis pathway in A549/PTX cells (Fig. 2C), while Gene Ontology (GO) enrichment analysis indicated modulation of serine hydrolase activity, implicating the combination of anlotinib and PTX had an impact on the serine metabolic pathway (Fig. 2D). These findings demonstrate that the combination treatment influences both glycolytic flux and serine-related metabolic processes in A549/PTX cells.

The metabolic product of glycolysis, 3-phosphoglycerate (3-PG), enters the SSP through the enzyme PHGDH and is ultimately converted into serine. Throughout this transformation, tumor cell glucose shifts from energy generation to biosynthesis, a pivotal step in facilitating cancer cell rapid growth (Fig. 2E). Based on the transcriptomic data, we subjected the pivotal genes implicated in the glycolytic process and serine metabolism to a cluster heatmap assessment. The findings revealed that the majority of these genes experienced a considerable reduction in expression within the combination therapy cohort (Fig. 2F). Notably, the PHGDH gene exhibited an especially significant decrease (Fig. 2G).

Additionally, we found that besides PHGDH, two other key SSP enzymes **–** PSAT1 and PSPH **–** were also upregulated in A549/PTX cells (Fig. 2H), suggesting that the SSP is activated in A549/PTX cells, which may contribute to PTX resistance in these cells. Consequently, inhibiting the SSP could represent a novel therapeutic strategy to overcome PTX resistance in NSCLC cells. The combination of anlotinib and PTX inhibited the SSP in A549/PTX cells, as evidenced by the significant downregulation of key SSP proteins (Fig. 2I) and a reduction in the level of its downstream product, serine (Fig. S1A). To further validate that inhibiting SSP in A549/PTX cells can reverse PTX resistance, we employed either the PHGDH inhibitor NCT-503 [24] or transfection with PHGDH-targeting siRNA in A549/PTX cells as positive controls for our experiment. Both NCT-503 (Fig. 2J-K) and siPHGDH (Fig. S2) suppressed the SSP and inhibited the proliferation activity of A549/PTX cells.

Research has shown that PHGDH-activated SSP is vital in governing the glycolytic process to meet the rapid proliferation demands of cancerous cells [25]. Our research revealed that A549/PTX cells exhibit enhanced glycolytic activity compared to A549 cells, primarily characterized by increased glucose uptake and lactate secretion. This enhanced activity enables A549/PTX cells to absorb more nutrients from their environment, thereby supporting their rapid proliferation. The combination of anlotinib and PTX effectively inhibits the SSP in A549/PTX cells, leading to a reduction in their glycolytic capacity and ultimately resulting in an insufficient energy for these cells (Fig. 2L-N). In summary, our experiments indicate that the activation of SSP is implicated in mediating PTX resistance in NSCLC, and the combination of anlotinib and PTX can suppress SSP in A549/PTX cells and impair their glycolytic ability.

### SSP inhibition suppresses the proliferation of A549/PTX cells by disrupting AKT/ERK signaling pathway

**Fig. 3:**
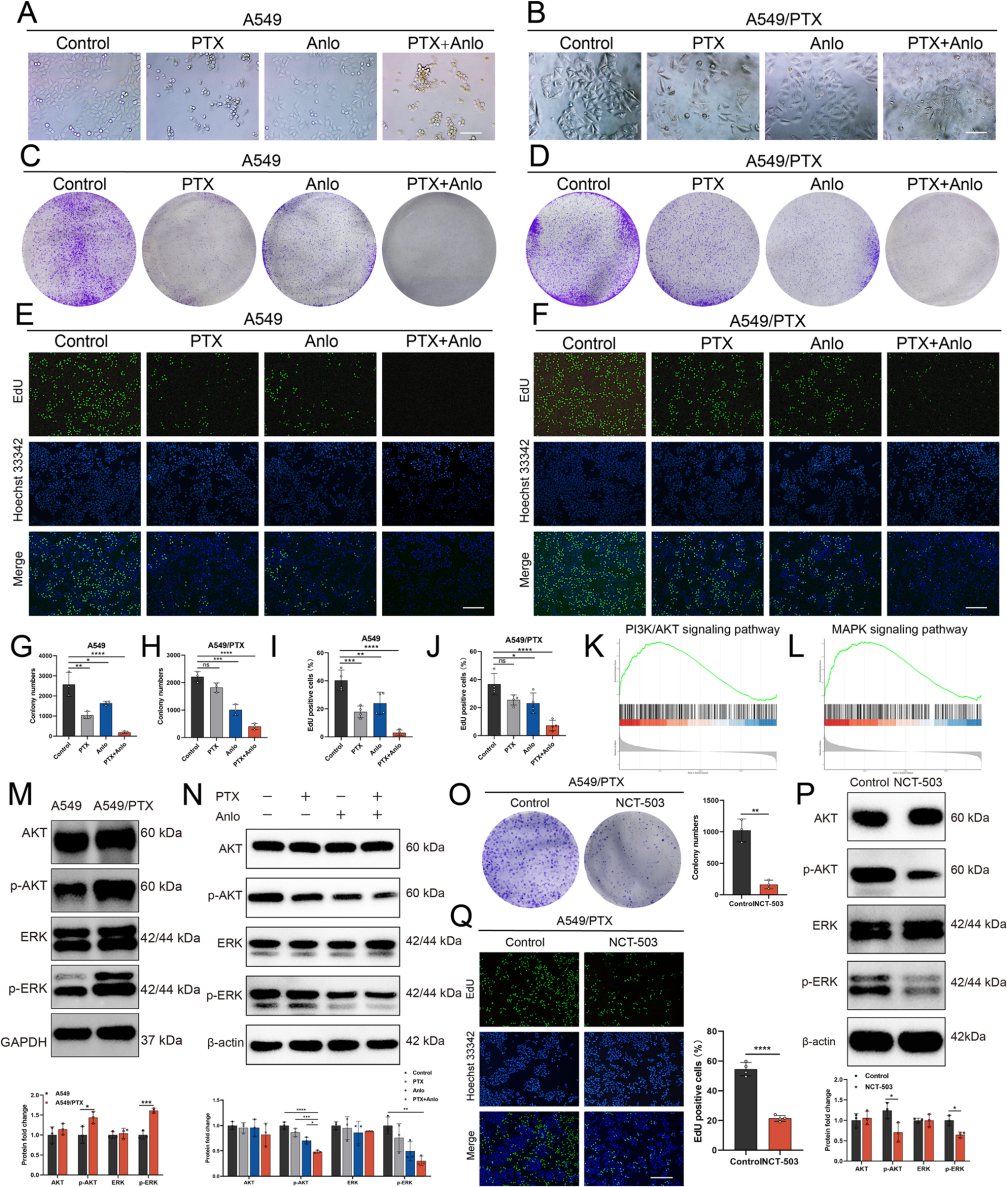
The combination of anlotinib and paclitaxel inhibits the SSP of A549/PTX cells resulting in impaired cell proliferation activity by disrupting AKT/ERK signaling pathway. A549 and A549/PTX cells were treated with PTX (6 μM), anlotinib (2 μM), or a combination of both for a duration of 72 h. Following treatment, **A**-**B** Microscopic observations (scale bar: 50 μm), **C**-**D**, **G**-**H **Colony formation assays (*n* = 3), and **E**-**F**, **I**-**J** EdU assays (scale bar: 200 μm; *n* = 3) were performed. **K**-**L** GSEA analysis between control group and combination treatment group. **M** The differences expression of AKT/ERK signaling proteins in A549 and A549/PTX cells were measured by western blotting. **N** Western blotting was used to analyze the expression of AKT/ERK signaling proteins in A549/PTX cells after treatment with PTX (6 μM), anlotinib (2 μM), or their combination for 24h. A549/PTX cells were treated with NCT-503 (22 μM) for 24 h, after which **O** colony formation assays (*n* = 3), **P** western blotting for AKT/ERK signaling proteins, and **Q** EdU assays (scale bar: 200 μm; *n* = 3) were conducted

The activated SSP is significantly associated with the rapid proliferation of cancer cells [26]. However, the specific mechanism by which anlotinib combined with PTX inhibits the SSP pathway in A549/PTX cells and affects their proliferative activity remains unclear. To address this issue, we first observed the morphology of A549 and A549/PTX cells following individual or combination treatment with PTX and anlotinib under high-power microscopy. Following combination treatment, both A549 cells and A549/PTX cells exhibited shrinkage and entered an apoptotic state (Fig. 3A-B). Furthermore, PTX monotherapy significantly inhibited the clonogenic ability and DNA replication activity of A549 cells but showed no obvious effect on A549/PTX cells. Notably, when anlotinib is combined with PTX, it not only restores the sensitivity of A549/PTX cells to PTX treatment but also further enhances its inhibitory effect on A549 cells (Fig. 3C-J). AKT and ERK serve as the primary effectors in the PI3K/AKT and MAPK signaling cascades, respectively [27]. Extensive preclinical and clinical research suggests that the activation of the AKT/ERK axis contributes to tumor growth and the development of resistance to chemotherapy [28, 29]. In this study, we investigated the impact of inhibiting SSP on the AKT/ERK signaling cascade. Initially, we conducted a gene set enrichment analysis (GSEA) and discovered a substantial reduction in gene expression associated with the PI3K/AKT and MAPK pathways, which are linked to cell proliferation, in A549/PTX cells post-combination therapy (Fig. 3K-L). Moreover, when compared to A549 cells, A549/PTX cells exhibited a marked increase in the levels of the key proteins p-AKT and p-ERK, suggesting that the AKT/ERK signaling pathway is indeed activated in A549/PTX cells (Fig. 3M). The combination therapy had a notable impact on lowering the levels of p-AKT and p-ERK in A549/PTX cells, indicating that the AKT/ERK signaling pathway was inhibited by the combination treatment (Fig. 3N). Furthermore, treatment with either the positive control NCT-503 (Fig. 3O-Q) or siPHGDH (Fig. S3) suppressed the growth of A549/PTX cells and impaired the AKT/ERK signaling pathway. These insights imply that the combination of anlotinib and PTX suppresses the SSP of A549/PTX cells, effectively blocking the AKT/ERK signaling pathway and hindering cell proliferation.

### SSP inhibition downregulates P-gp expression and impairs its transporter function in A549/PTX cells

**Fig. 4:**
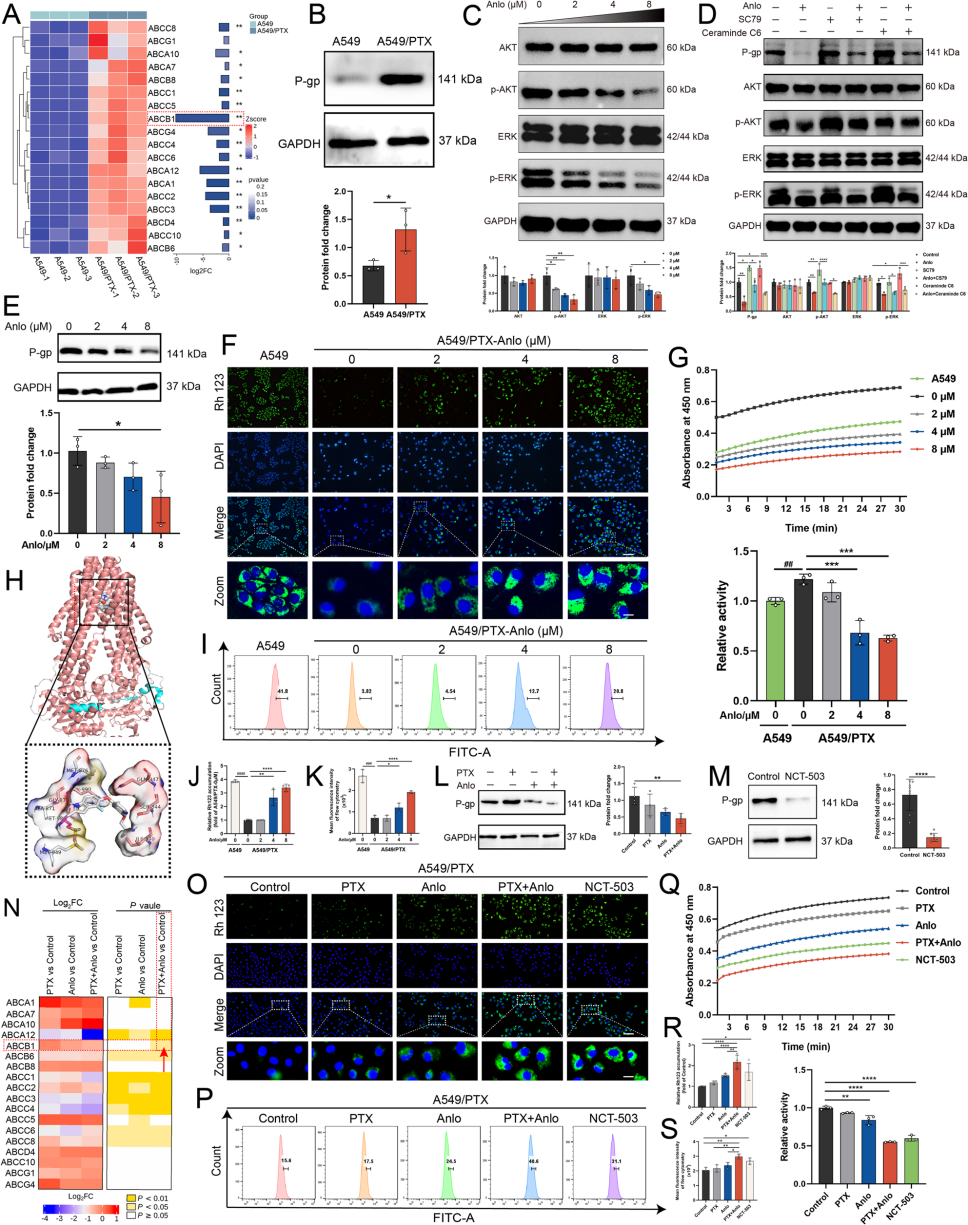
The combination of anlotinib and paclitaxel reduces P-gp expression and transport function in A549/PTX cells by inhibiting SSP. **A** Cluster heatmap analysis of ABC transporter family genes. **B** Western blotting of P-gp in A549 and A549/PTX cells. **C** Effects of anlotinib alone on the expression of AKT/ERK pathway-related proteins in A549/PTX cells (*n* = 3). **D** Western blotting was performed to examine the expression of proteins in the AKT/ERK pathways and P‑gp following treatment with anlotinib (8 μM) alone or in combination with either SC79 (an Akt activator, 10 μM) or C6-ceramide (an ERK1/2 activator, 10 μM) (*n* = 3). **E** Western blotting was performed to examine the effect of varying concentrations of anlotinib on P-gp expression in A549/PTX cells. **F**, **J** Fluorescence microscopy and **I**, **K** flow cytometry to detect Rh123 accumulation (*n* = 3). **G** Anlotinib suppress the activity of the PHGDH enzyme (*n* = 3). **H** Molecular docking analysis of anlotinib with the P-gp protein (PDB: 6QEX). **L** Western blotting was performed to detect the difference in P-gp expression after treatment of A549/PTX cells with PTX (6 μM), anlotinib (2 μM), or their combination. **M** Western blotting was used to examine the change in P-gp expression after NCT-503 (22 μM) treatment in A549/PTX cells. **N** Cluster heatmap analysis of ABC transporter family genes among different comparison groups of A549/PTX cells. **O**, **R** Fluorescence microscopy and **P**, **S** flow cytometry to detect the difference in Rh123 accumulation in A549/PTX cells treated with different drugs (*n* = 3). **Q** The combination of anlotinib and paclitaxel suppresses the activity of the PHGDH enzyme (*n* = 3). Scale bar: 100 μm ; Zoom scale bar: 20 μm

P-gp is encoded by the ABCB1 gene of the B subfamily. This protein relies on ATP hydrolysis for energy and actively transports intracellular drugs out of the cell, thereby reducing their efficacy. Its high expression is a common reason for PTX resistance in NSCLC [5]. Previous studies have demonstrated that the AKT/ERK signaling pathway can regulate the expression of the P-gp protein [30]. Given this, we hypothesized that the combination of anlotinib and PTX inhibits SSP in A549/PTX cells, leading to impaired transmission of the AKT/ERK signaling pathway and a reduction in ATP content. This effect may also inhibit the expression and transport function of P-gp, thereby overcoming PTX resistance in NSCLC. To verify our hypothesis, we initially conducted a transcriptomic analysis, which revealed that genes associated with the ABC family were significantly overexpressed in A549/PTX cells, with ABCB1 emerging as the most prominent (Fig. 4A). Furthermore, the P-gp encoded by this gene was also found to be highly expressed in A549/PTX cells (Fig. 4B). Anlotinib formed stable interactions with amino acid residues in the P-gp substrate-binding domain (Fig. 4H) and consequently downregulates P‑gp protein expression in A549/PTX cells (Fig. 4E) by inhibiting the activation of the AKT/ERK signaling pathway (Fig. C-D). Rh123, a fluorescent substrate of P-gp, directly reflects the transport function of P-gp based on its accumulation in cells [31]. By detecting the changes in Rh123 accumulation in NSCLC cells using fluorescence microscopy and flow cytometry, the results revealed that the levels of Rh123 accumulation in A549/PTX cells were markedly lower compared to the accumulation observed in A549 cells. After treatment with anlotinib, the accumulation of Rh123 in A549/PTX cells significantly increased (Fig. 4F, I-K). Furthermore, anlotinib inhibited the activity of the PHGDH enzyme (Fig. 4G), which suggests that its downregulation of P-gp protein expression and inhibition of SSP function are related to this effect. These results indicate that anlotinib suppresses P-gp expression and function by inhibiting the activity of SSP, suggesting it is a potent P-gp inhibitor candidate. This finding supports our further exploration of the effects of combining anlotinib with PTX on the expression and function of P-gp protein. Firstly, in the A549/PTX cells, transcriptomic analysis revealed that the combination of anlotinib and PTX significantly down-regulated the expression of the ABCB1 gene (Fig. 4N). Furthermore, western blotting results indicated that the combined effects of anlotinib and PTX, as well as NCT-503 as a single agent, significantly inhibited the expression of P-gp protein in A549/PTX cells (Fig. 4L-M), which correlated with the inhibition of PHGDH enzyme activity (Fig. 4Q). Consistently, these treatments also markedly enhanced the intracellular accumulation of Rh123 (Fig. 4O-P, R-S). This effect was mirrored by PHGDH knockdown, where transfection with siPHGDH similarly reduced P-gp levels and increased Rh123 accumulation (Fig. S4). In conclusion, the synergistic combination of anlotinib and PTX can effectively reverse PTX resistance in NSCLC by inhibiting the SSP of A549/PTX cells, thereby down-regulating the expression of P-gp protein and inhibiting its transport function.

### Inhibiting SSP blocks the EMT process in A549/PTX cells

**Fig. 5:**
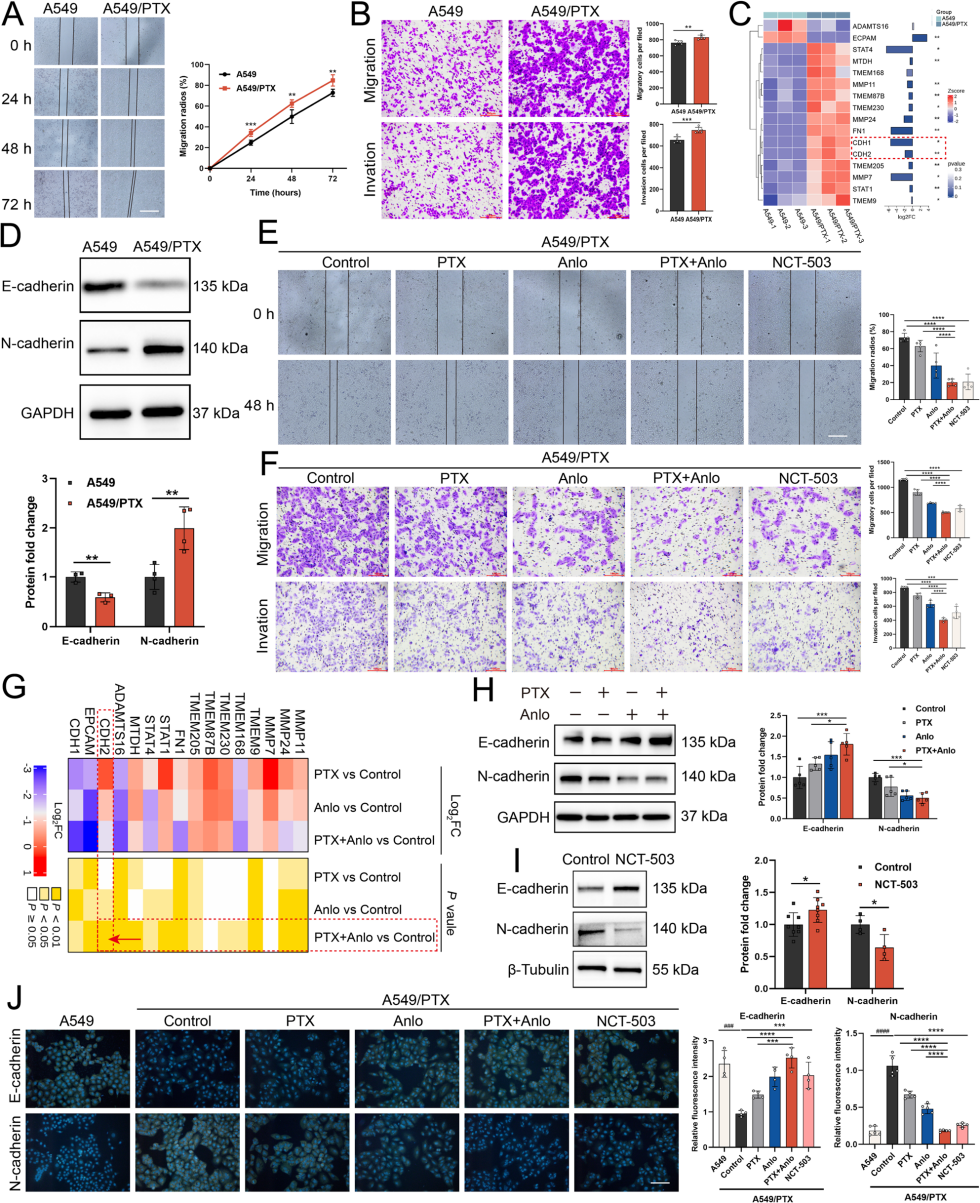
The combination of anlotinib and paclitaxel reverses the EMT process in A549/PTX cells by inhibiting SSP. **A** Wound-healing migration assay (scale bar: 200 μm; *n* = 4) and **B** transwell assay (*n* = 5) for A549 and A549/PTX cells. **C** Cluster heatmap analysis of EMT-related genes. **D** Western blotting of EMT markers in A549 and A549/PTX cells. **E** Wound-healing migration assay (scale bar: 200 μm; *n* = 5) and **F** transwell assay (*n* = 4) in A549/PTX cells treated with different drugs. **G** Cluster heatmap analysis of EMT-related genes in different comparison groups of A549/PTX cells. **H**-**I** Western blotting and **J** immunofluorescence of EMT markers in A549/PTX cells following drug treatments (scale bar: 200 μm)

EMT is also recognized as a significant contributor to PTX resistance in NSCLC and has a strong correlation with SSP. The activation of the SSP or the heightened expression of PHGDH, PSAT1, and PSPH contributes to the stimulation of the EMT process, thereby promoting cancer cell metastasis and drug resistance [6, 32]. Consequently, this study also investigates the impact of anlotinib in combination with PTX on the EMT process in A549/PTX cells following the inhibition of SSP in these cells. As demonstrated by wound-healing migration assay (Fig. 5A) and transwell assay (Fig. 5B), A549/PTX cells exhibited stronger migration and invasion capabilities than A549 cells. To elucidate the potential molecular basis of these changes, we initially conducted a transcriptomic analysis to explore changes in the expression of genes linked to the EMT process. Notably, CDH2 (encoding N-cadherin, a key mesenchymal marker) was significantly upregulated in A549/PTX cells; in contrast, although CDH1 (encoding E-cadherin, an epithelial marker) initially appeared elevated in transcriptomic data (Fig. 5C), further validation at the protein level revealed both a decrease in E-cadherin expression and an increase in N-cadherin expression in A549/PTX cells (Fig. 5D). These findings confirm EMT activation in A549/PTX cells, consistent with their enhanced migratory and invasive capabilities. The combination of anlotinib with PTX significantly reduced the migration and invasion capabilities of A549/PTX cells (Fig. 5E-F). To clarify the specific mechanism behind this combination treatment, transcriptomic analysis revealed that anlotinib and PTX significantly down-regulated genes linked to the EMT process (Fig. 5G). Furthermore, we scrutinized the expression levels of key EMT markers **–** E-cadherin and N-cadherin **–** via western blotting and immunofluorescence techniques. Our results revealed a noteworthy shift: the combination therapy increased E-cadherin levels, while suppressing N-cadherin expression. A similar trend was observed in A549/PTX cells following treatment with NCT-503 (Fig. 5H-J) or transfection with siPHGDH (Fig. S5), indicating reversal of the EMT process. These experiments indicate that the combination of anlotinib and PTX can inhibit the EMT process by suppressing SSP in A549/PTX cells, thereby reducing cell migration and invasion capabilities and overcoming PTX resistance in NSCLC.

### Suppressing SSP triggers the mitochondrial apoptosis pathway in A549/PTX cells and induces cell apoptosis

**Fig. 6:**
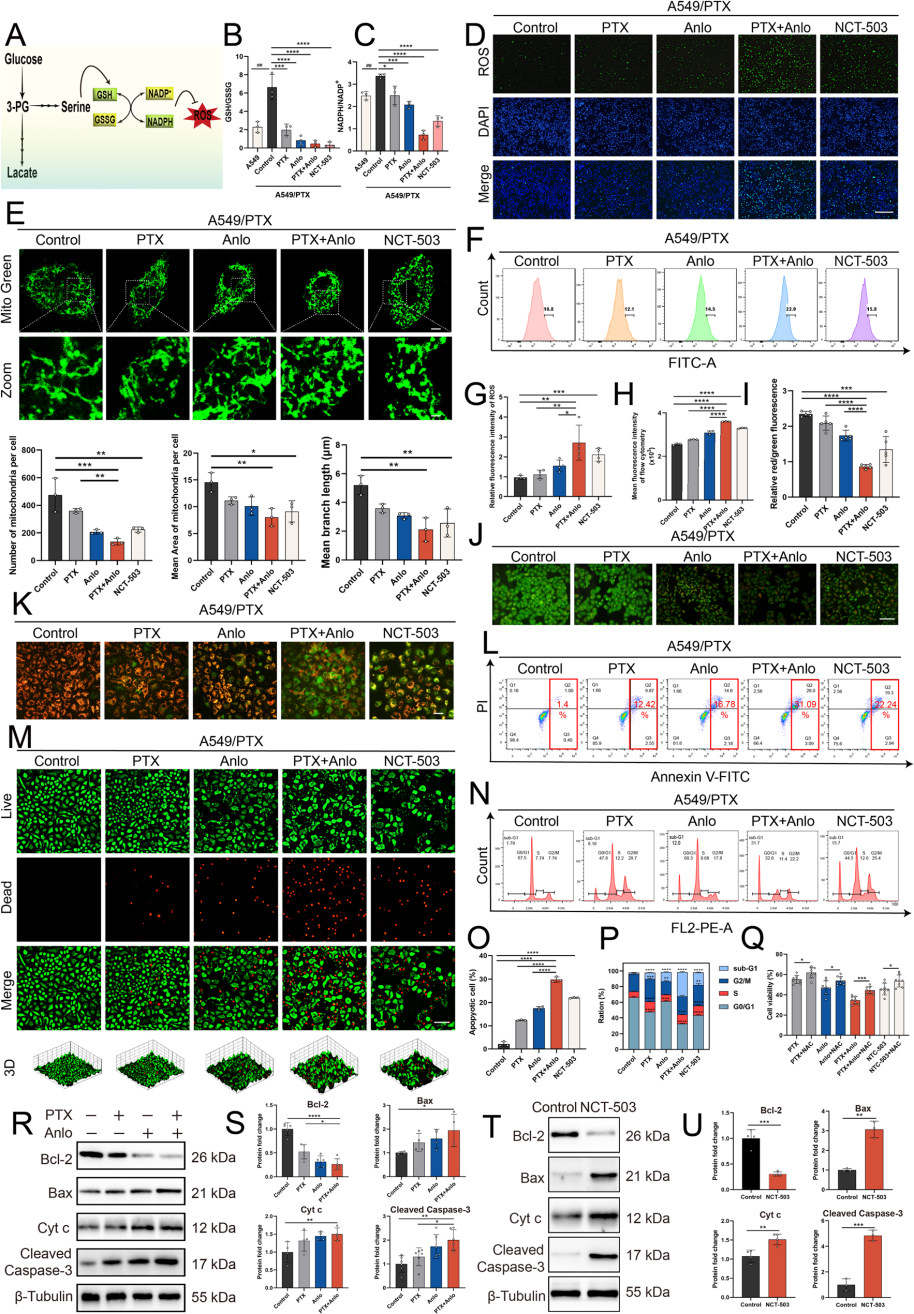
The combination of anlotinib and paclitaxel activates the mitochondrial apoptotic pathway and induces apoptosis by inhibiting SSP in A549/PTX cells. **A** The simplified schematic diagram shows that SSP maintains the cellular redox homeostasis by regulating GSH and NADPH regeneration to resist ROS. **B** The ratios of GSH/GSSG (*n* = 3) and **C** NADPH/NADP^+^ (*n* = 3) were measured after various drug treatments in A549/PTX cells, while A549 cells remained untreated. PTX (6 μM), anlotinib (2 μM), PTX + anlotinib, and NCT-503 (22 μM) were applied to A549/PTX cells for 48 h. Then, **D**, **G** ROS fluorescence microscopy observation (scale bar : 200 μm), **E** mitochondrial morphology detection and quantitative analysis (scale bar: 100 μm, Zoom image scale bar: 20 μm), **F**, **H** ROS flow cytometry detection (*n* = 3), **J** AO staining experiment (scale bar: 200 μm), **I**, **K** mitochondrial membrane potential detection (scale bar:50 μm), **L**, **O** Annexin V-FITC/PI staining (*n* = 3), **M** AM/PI staining experiment (scale bar:100 μm), and **N**, **P** cell cycle flow cytometry detection (*n* = 3) were performed. **Q** MTT assay (*n* = 8) was conducted to evaluate the protective effect of NAC (20 mM) against cell apoptosis caused by various drug treatments. Western blotting was performed to detect changes in mitochondrial apoptosis-associated proteins in A549/PTX cells exposed to **R**-**S** PTX (6 μM) and/or anlotinib (2 μM), or **T**-**U**. NCT-503 (22 μM)

SSP utilizes synthetic GSH and NADPH as antioxidants to combat oxidative stress induced by increased ROS. This mechanism helps maintain cellular redox balance and contributes to chemotherapy resistance in tumors (Fig. 6A). In our study, A549/PTX cells showed higher GSH/GSSG and NADPH/NADP^+^ ratios than A549 cells, suggesting that SSP pathway activation enhances GSH and NADPH production to mitigate oxidative stress. Both combination treatment and NCT-503 monotherapy reduced these ratios, thereby suppressing SSP. This suppression elevated ROS levels, inducing oxidative stress (Fig. 6B-D, F-H). Mitochondrial morphology and function are closely interconnected. Elevated ROS levels lead to oxidative stress, causing mitochondrial damage and dysfunction, which ultimately induces cell cycle arrest and may trigger apoptosis [33]. Both combination therapy and NCT-503 monotherapy altered mitochondrial morphology in A549/PTX cells (Fig. 6E) and significantly decreased mitochondrial membrane potential (Fig. 6I and K). Observations of apoptosis through acridine orange (AO) staining indicated that anlotinib further enhanced apoptosis in A549/PTX cells following PTX treatment, while NCT-503 also induced cell apoptosis (Fig. 6J). The results from flow cytometry utilizing Annexin V-FITC/PI double staining (Fig. 6L and O) and live-dead staining (Fig. 6M) experiments corroborated these findings. It is noteworthy that the antioxidant N-acetylcysteine (NAC) can partially reverse the cell apoptosis induced by the simultaneous administration of both drugs, further indicating that the increase in ROS in A549/PTX cells plays a crucial role in inducing apoptosis (Fig. 6Q). Additionally, the combination treatment and NCT-503 monotherapy also cause A549/PTX cells to be arrested in the G1 phase (Fig. 6N and P). The increase in ROS can activate the mitochondrial apoptosis pathway [33]. We investigated the expression of proteins associated with this pathway following the combination treatment. The results indicated that the combination of anlotinib and PTX significantly decreased Bcl-2 in A549/PTX cells compared to the use of PTX alone, while simultaneously increasing the expression of Bax, cytochrome c (Cyt c), and Cleaved Caspase-3 (Fig. 6R-S). Additionally, following NCT-503 treatment of A549/PTX cells, changes in mitochondrial apoptosis-related protein expression mirrored those observed with the combination therapy (Fig. 6T-U). These results demonstrate that the combination of anlotinib and PTX can effectively inhibit the SSP pathway in A549/PTX cells. This inhibition will promote the production of ROS and trigger the mitochondrial apoptosis cascade, ultimately promoting cell death and overcoming PTX resistance in NSCLC.

### Combination of anlotinib with paclitaxel impedes the growth of xenografted A549/PTX NSCLC tumors in nude mice

**Fig. 7:**
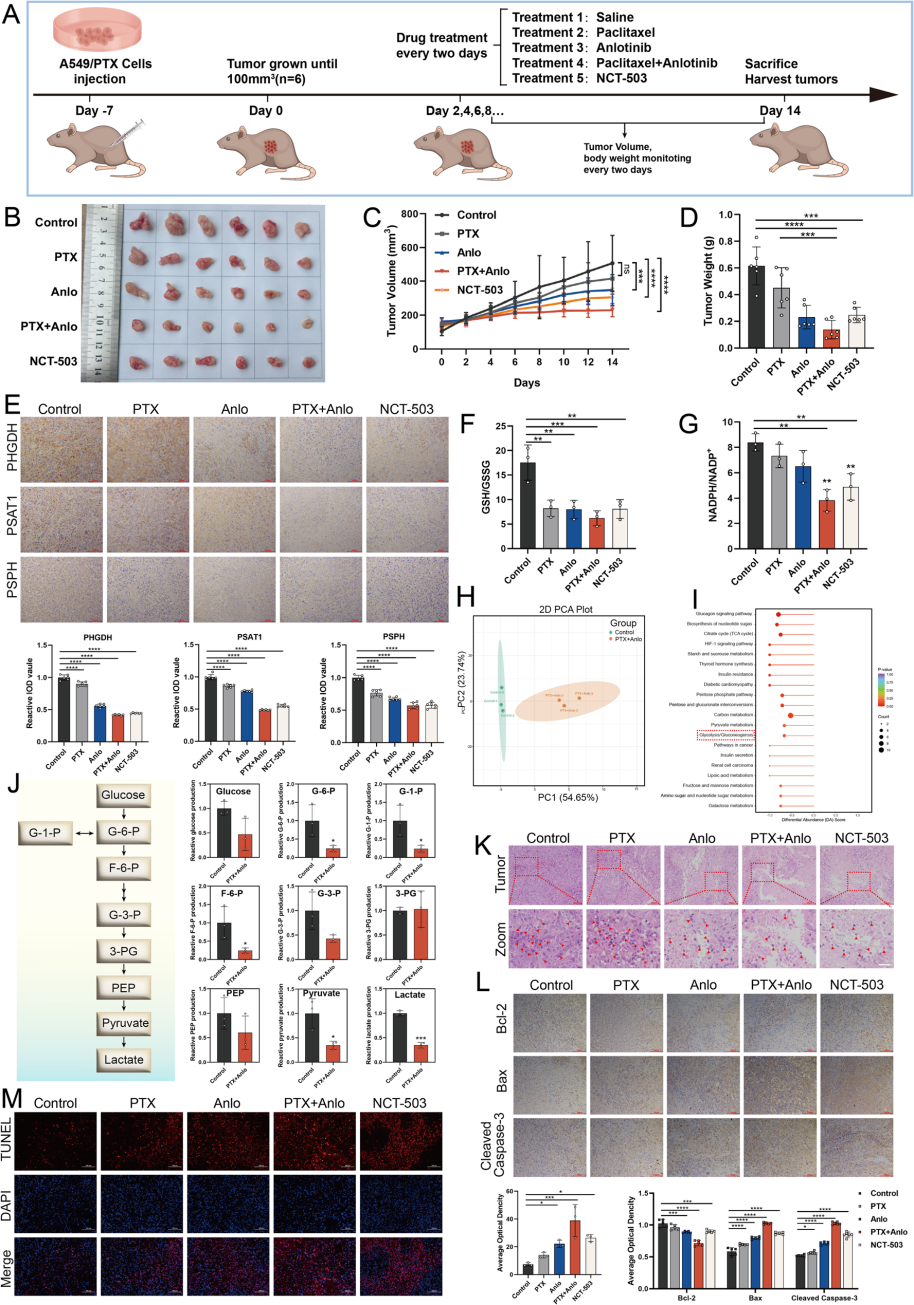
The combination of anlotinib and paclitaxel inhibits growth of A549/PTX xenografts in nude mice. **A** *In vivo* experimental design schematic. **B** Representative tumor photographs from each group(*n* = 6). **C** Tumor volume growth curve (measured every 2 days;* n* = 6). **D** Final tumor weights after 14 days treatment (*n* = 6 ). E Immunohistochemical staining of key SSP proteins in tumor tissues (*n* = 5). **F-G** GSH/GSSG (n = 3) and NADPH/NADPH^+^ (*n* = 3) ratios in tumor tissues. **H** PCA of serum metabolites: control vs combination. **I** DA scores for KEEG pathways between the control and combination treatment group. **J** The content levels of main substances in the glycolysis pathway (*n* = 3), including: glucose, glucose-6-phosphate (G-6-P), glucose-1-phosphate (G-1-P), fructose-6-phosphate (F-6-P), glyceraldehyde-3-phosphate (G-3-P), 3-phosphoglyceric acid (3-PG), phosphoenolpyruvic acid (PEP), pyruvate, lactate, were downregulated after combination treatment. **K** H&E staining of tumor tissue (Zoom scale bar: 50 μm). **L** Immunohistochemical analysis reveals the expression levels of apoptosis-associated proteins in tumor tissue. **M** TUNEL staining of tumor tissue (*n* = 3)

**Fig. 8:**
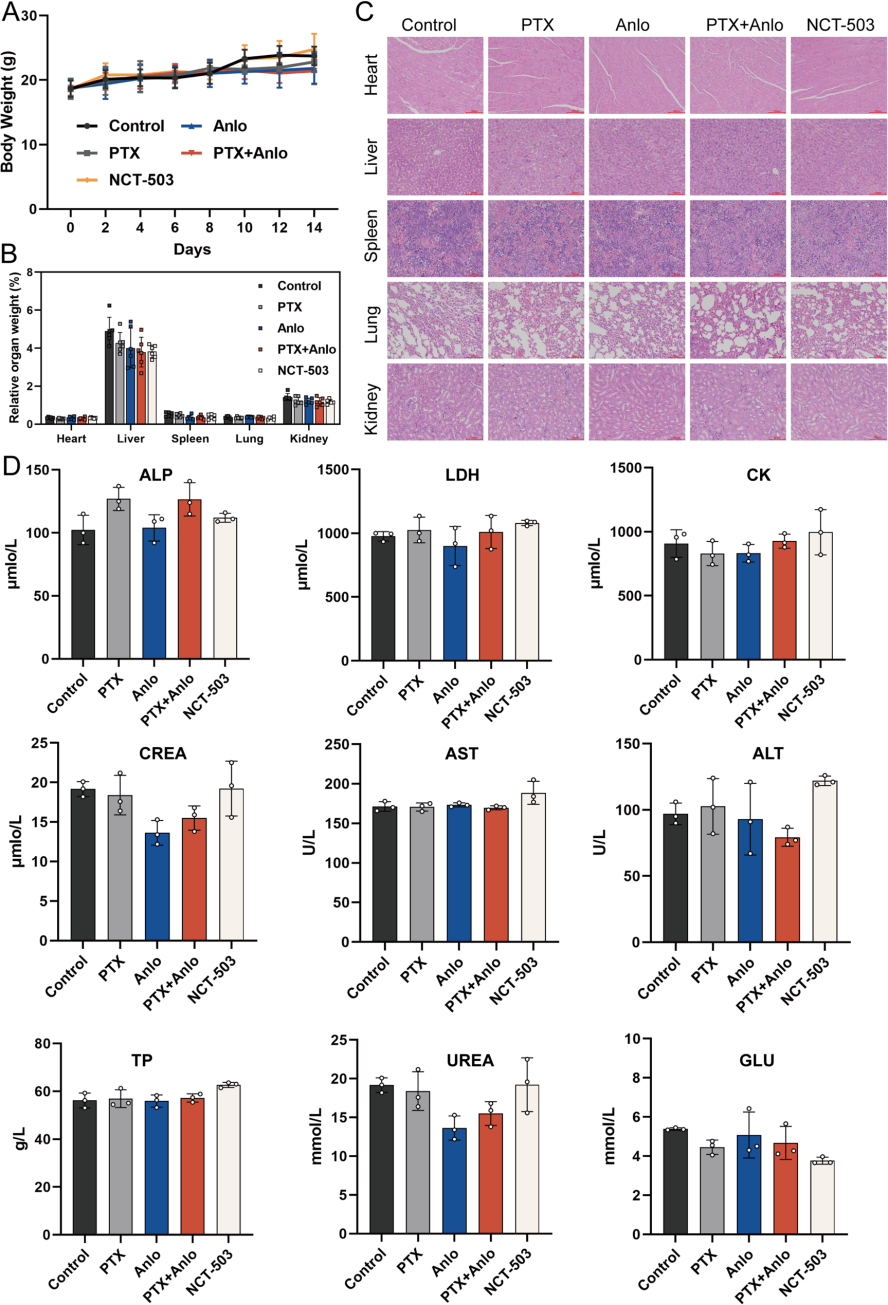
The combination treatment of anlotinib and paclitaxel exhibits favorable safety in A549/PTX xenograft models. **A** Body weights of the mice were recorded every two days ( *n* = 6). **B** Organ indices across treatment groups (*n* = 6). **C** H&E staining of major organs. **D** A serum biochemical analysis was conducted on nude mice from distinct treatment groups (*n* = 3), including indicators: alkaline phosphatase (ALP), lactate dehydrogenase (LDH), creatine kinase (CK), creatinine (CREA), aspartate aminotransferase (AST), alanine aminotransferase (ALT), total protein (TP), urea nitrogen (UREA), and blood glucose (GLU)

To evaluate the effectiveness of anlotinib combined with PTX on the *in vivo* proliferation of A549/PTX cells, a nude mouse xenograft model was established by subcutaneous inoculation. Drugs were administered every two days, with monitoring of body weight and tumor size (Fig. 7A). Compared to PTX monotherapy, the combination treatment demonstrated significantly enhanced tumor growth inhibition. Similarly, NCT-503 alone also exhibited significant antitumor activity, albeit less pronounced than the combination(Fig. 7B - D). No significant differences in body weight(Fig. 8A) or organ coefficient analysis (Fig. 8B) were observed among treatment groups. Importantly, histological and biochemical assessments confirmed that the combination regimen was well tolerated, showing neither structural organ damage nor impairment of hepatic/renal function (Fig. 8C-D). These results indicate that the combination treatment not only exhibits a potent anti-tumor effect but also demonstrates good safety profiles.

To investigate the effect of combination therapy on the SSP pathway in A549/PTX tumor tissue from nude mice, we analyzed expression changes of key SSP proteins and quantified the serine levels within the tumor tissues. Relative to the control, the combination treatment and NCT-503 groups demonstrated markedly decreased levels of PHGDH, PSAT1, and PSPH proteins (Fig. 7E), along with a reduction in serine content (Fig. S1B). These results demonstrate that the anlotinib-PTX combination therapy effectively inhibits SSP in tumor tissues *in vivo*. The inhibition of SSP led to lower levels of GSH and NADPH in the tumor tissues, ultimately causing an imbalance in redox homeostasis, which aligns with the results obtained from *in vitro* studies (Fig. 7F-G). To further analyze the effect of SSP on PTX resistance, we collected serum from nude mice in the Control group and the combination treatment group for LC-MS targeted metabolomics analysis. Principal component analysis (PCA) revealed that the combination treatment altered the metabolic status of the nude mice(Fig. 7H). Differential abundance scores (DA scores) for KEGG pathways indicated combination therapy impacted glycolysis/gluconeogenesis (Fig. 7I). The products of glycolysis enter the SSP through PHGDH, and the inhibition of SSP can diminish glycolytic capacity [25]. In our study, we found that the combination treatment downregulated the levels of most glycolytic metabolites, with significant differences observed in G1P, G6P, F6P, pyruvate, and lactate(Fig. 7J). Furthermore, H&E staining (Fig. 7K) and TUNEL staining (Fig. 7M) results indicated that the combination therapy induced significant cell death, mediated through activation of the Bcl-2/Bax/Cleaved Caspase-3 apoptotic pathway in tumor tissues (Fig. 7L). Notably, although anlotinib is a classic anti-angiogenic drug, the enhanced efficacy of its combination with paclitaxel did not primarily stem from augmented anti-angiogenesis (Fig. S6). Collectively, these findings demonstrate that anlotinib combined with PTX synergistically inhibits the growth of PTX-resistant NSCLC xenograft tumors by suppressing the SSP in tumor cells *in vivo*.

## Discussion

PTX is a first-line chemotherapy drug for advanced NSCLC. However, the development of PTX resistance remains a significant challenge in NSCLC chemotherapy, leading to treatment failure and high mortality rates [34]. Therefore, exploring the molecular mechanisms underlying PTX resistance is crucial for identifying novel therapeutic strategies to overcome this limitation.

Serine metabolism constitutes a critical metabolic hub within tumors, supplying essential macromolecular substances that support their growth and rapid proliferation, as well as important cofactors necessary for maintaining redox balance through specific metabolic reprogramming [35]. Convincing evidence suggests that the metabolic reprogramming of serine metabolism, particularly the SSP, is linked to drug resistance in various cancers [36]. SSP elevation in lung cancer is a common phenomenon [9], with increased expression of key enzymes, including PHGDH [37], PSAT1 [38], and PSPH [39], playing a crucial role in promoting aggressive progression of lung cancer and treatment resistance. These enzymes are capable of interacting with various signaling pathways, thereby promoting the development of lung cancer. Research has indicated that reducing exogenous serine intake or inhibiting the production of endogenous serine can lead to decreased tumor growth and improves survival for individuals with tumors [13]. Therefore, targeting the SSP may represent a novel therapeutic strategy to overcome PTX resistance in NSCLC.

Anlotinib is a novel orally administered multi-target receptor tyrosine kinase inhibitor. Its mechanism involves the simultaneous inhibition of key targets including VEGFR and PDGFR, as well as downstream signaling pathways such as PI3K/AKT and MAPK/ERK. This multi-targeted action effectively suppresses tumor angiogenesis and promotes vascular normalization. These effects collectively enhance the intratumoral delivery and distribution of various anti-tumor agents while modulating the immunosuppressive tumor microenvironment, leading to broader and more sustained therapeutic efficacy compared to single-targeted therapies [16]. Furthermore, anlotinib inhibits tumor proliferation [40], induces oxidative stress and apoptosis [41], and reverses human ABCB1-mediated multidrug resistance [42]. It is currently approved for third-line treatment of NSCLC. Given that paclitaxel resistance represents a major clinical challenge, combination strategies utilizing anlotinib to reverse resistance and restore tumor chemosensitivity hold significant clinical value and application prospects.

Clinical trials have established the value of anlotinib in treating NSCLC. Both the Phase II ALTER0302 [43] and Phase III ALTER0303 [44] studies demonstrated that, compared with placebo, anlotinib significantly improved objective response rate (ORR) and disease control rate (DCR), while also prolonging progression-free survival (PFS) and overall survival (OS) in patients with advanced NSCLC. Subsequent real-world studies have further confirmed its efficacy and safety, particularly in patients intolerant to chemotherapy [17, 45]. In terms of combination therapy, anlotinib paired with chemotherapy (e.g., erlotinib [46], S-1 [47] ), radiotherapy [48], or immune checkpoint inhibitors [49] has shown promising efficacy. Notably, its combination with taxane-based agents, such as docetaxel, demonstrates a synergistic antitumor effect. Studies including ALTER‑L016 [50], ALTER‑L018 [51], and ChiCTR1800020011 [52] have confirmed that this regimen significantly prolongs median PFS, improves ORR and DCR, and maintains manageable safety compared with chemotherapy alone.

PHGDH, as the rate-limiting enzyme in the first step of the SSP, catalyzes the conversion of 3-PG into 3-phosphohydroxypyruvate (3-PHP). Its activity critically governs the metabolic flux through SSP [53]. Inhibiting PHGDH directly blocks the production of 3-PHP, leading to functional idleness of downstream enzymes PSAT1 and PSPH due to substrate deprivation. This results in a physical interruption of the entire SSP flux, thereby impairing the synthesis of serine, glycine, and one-carbon units. Consequently, cells experience a crisis in nucleotide synthesis and methyl donor availability, which functionally suppresses proliferation in SSP-dependent contexts [54] .Furthermore, as a major source of one-carbon units, PHGDH plays a key role in regulating redox homeostasis within one-carbon metabolism. PHGDH inhibition sharply reduces NADPH production, disrupting the GSH regeneration system and leading to metabolic disturbances such as oxidative stress [54]. This metabolic pressure activates stress-responsive signaling pathways, including the integrated stress response centered on ATF4, triggering adaptive transcriptional reprogramming and profound epigenetic remodeling. Due to shortages in one-carbon units and α-ketoglutarate, histone and DNA methylation patterns become dysregulated, globally affecting gene expression [55, 56]. Ultimately, these direct metabolic disruptions, combined with multilayered feedback—including transcriptional downregulation, protein modifications, and pathway reprogramming—create a “downward spiral” that systematically dismantles SSP functionality. Cells reliant on this pathway consequently undergo growth arrest or death due to compounded metabolic, oxidative, and replicative stresses.

The high expression of PHGDH is closely associated with tumorigenesis, drug resistance, and poor prognosis, making it a primary target in current drug development targeting the SSP [54]. Research demonstrates that PHGDH inhibition effectively reverses drug resistance and enhances treatment efficacy. For instance, in erlotinib-resistant lung adenocarcinoma, suppression of PHGDH via siRNA or the inhibitor NCT‑503 restores sensitivity to erlotinib [37]. Moreover, PHGDH inhibitors combined with serine/glycine‑depleted medium suppress tumor growth by disrupting the synthesis of DNA, purines, and glutathione [57] .Additionally, synergistic effects have been observed when the PHGDH inhibitor NCT‑503 is combined with the pyruvate kinase M2 inhibitor PKM2‑IN‑1 in A549 cells, leading to further inhibition of proliferation, induction of cell‑cycle arrest, and modulation of signaling pathways such as AMPK/mTOR [58]. Combining SSP inhibitors with chemotherapy also shows promising synergy; for example, in bladder cancer, PHGDH inhibitors enhance the efficacy of gemcitabine/cisplatin [59, 60].Given that no known metabolic pathway can bypass PSAT1 or PSPH downstream of PHGDH [61], targeting these enzymes may yield effects similar to PHGDH inhibition. Future research may therefore focus on selecting targeted agents based on the expression profiles of SSP key enzymes across different tumor types.

In this study, we demonstrate that SSP plays a critical role in mediating resistance to PTX in NSCLC. SSP is activated in NSCLC PTX-resistant cells, promoting the rapid growth of tumor cells. The combination of anlotinib and PTX effectively inhibits the SSP in A549/PTX cells, resulting in a significant reduction in their proliferative capacity. To elucidate how SSP inhibition reduces cell proliferation, we conducted GSEA analysis and revealed that the dual drug combination significantly impacts the PI3K/AKT and MAPK signaling pathways, which are crucial for regulating cell proliferation. The core effector molecules of these pathways are commonly referred to as the AKT/ERK signaling axis. In A549/PTX cells, the AKT/ERK signaling pathway is activated. However, both anlotinib-PTX combination and PHGDH inhibition (via NCT-503 or siPHGDH) inhibit this activation. Furthermore, since SSP serves as a pivotal junction in the glycolytic pathway, it exerts regulatory effects on glycolysis. Previous studies by Zhang et al. demonstrated that inhibiting the key rate-limiting enzyme, PHGDH, in the SSP pathway leads to reduced glycolytic activity [25]. In our investigation, we observed that A549/PTX cells exhibit elevated glycolytic activity, which provides a survival advantage to the tumor cells. The inhibition of SSP by the combination of anlotinib and PTX diminishes the glycolytic capacity of A549/PTX cells, thereby restricting the energy supply to the tumor cells. In conclusion, our findings suggest that the combination of anlotinib and PTX inhibits SSP in A549/PTX cells, thereby obstructing the AKT/ERK signaling pathway and ultimately impairing the proliferative activity of drug-resistant cells.

The drug efflux mediated by P-gp is a well-established mechanism of PTX resistance in NSCLC. P-gp actively transports chemotherapy drugs out of cells through ATP hydrolysis, leading to a reduction in the effective intracellular concentration of these drugs [5]. Research has indicated that activation of the AKT/ERK signaling pathway can enhance P-gp expression and its transport function, which represents a potential strategy for reversing PTX resistance in NSCLC [62]. Our previous experiments confirmed that the combined administration of anlotinib and PTX in A549/PTX cells inhibits SSP, disrupts the conduction of the AKT/ERK signaling pathway, and decreases ATP levels. This suggests that the combination therapy may also overcome PTX resistance in NSCLC through modulation of P-gp expression and transport activity. P-gp is highly expressed in A549/PTX cells, a characteristic feature of drug resistance. Anlotinib binds to P-gp and dose-dependently downregulates its expression while limiting transport function. This indicates that anlotinib is a promising P-gp inhibitor, prompting us further exploration of its combined effects with PTX on P-gp in A549/PTX cells. Notably, our research demonstrated that the use of anlotinib in conjunction with PTX notably decreased the expression of P-gp in A549/PTX cells while also inhibiting its transport function. Furthermore, validation with NCT-503 and siPHGDH in A549/PTX cells definitively established that the observed inhibition was mediated by suppression of the SSP.

The relationship between EMT and PTX resistance in NSCLC is particularly significant. The hallmarks of EMT are reduced expression of E-cadherin and increased expression of N-cadherin. This shift in protein expression is closely related to the invasion, spread and reduced response to chemotherapy of cancer cells. Inhibition of EMT can restore the effectiveness of chemotherapy drugs in cancer treatment [6, 63]. Multiple studies have established a significant association between the SSP pathway and cancer metastasis. Key SSP enzymes including PHGDH [64], PSAT1 [38], and PSPH [65] show elevated expression levels that promote tumor metastasis. Furthermore, P-gp-overexpressing tumors frequently exhibit EMT activation [66], which in turn upregulates P-gp expression and activity [67]. This effect may be due to the coordinated regulation of shared signaling pathways, such as the Wnt/β-catenin, PI3K/AKT and MAPK signaling pathways. Taking these findings into account, we hypothesize that the combination of anlotinib and PTX overcomes PTX resistance in NSCLC by inhibiting the SSP in A549/PTX cells, potentially through suppression of the EMT process. Our findings demonstrate that EMT activation in A549/PTX cells enhances their migratory and invasive capacities. The combination of anlotinib with PTX as well as single-agent NCT-503 or siPHGDH reverses EMT by inhibiting SSP in A549/PTX cells, consequently attenuating the metastatic potential of resistant cells and overcoming PTX resistance in NSCLC.

It is widely recognized that SSP plays a key role in regenerating NADPH and producing GSH, thereby regulating cellular redox homeostasis [68]. Redox homeostasis is essential for both the initiation and advancement of tumors. Tumor cells frequently display higher redox levels than normal cells, allowing cancer cells to acquire resistance to drugs and enhance antioxidant mechanisms to counteract significant oxidative stress [69]. Research indicates that disrupting cellular redox homeostasis, particularly the imbalance of ROS/GSH, can lead to detrimental oxidation and chemical modifications of biological macromolecules, ultimately causing cell cycle arrest, inhibiting proliferation, and potentially inducing cell death [70]. In this study, we observed that the combination of anlotinib and PTX inhibited the SSP in A549/PTX cells, leading to significantly increased ROS levels and subsequent mitochondrial dysfunction. This impairment was characterized by abnormal mitochondrial morphology and loss of membrane potential. In NSCLC, elevated ROS stimulate mitochondrion-mediated apoptosis, evidenced by the anti-apoptotic protein Bcl-2 and an increase in the pro-apoptotic protein Bax. Furthermore, these elevated ROS levels also promoted outer mitochondrial membrane pore opening, resulting in Cyt c release and subsequent Caspase-3 activation. To verify the occurrence of cell apoptosis, we tested the level of Cleaved Caspase-3 because it serves as a direct indicator of Caspase-3 activation. In summary, our results showed that anlotinib plus PTX suppressed SSP in A549/PTX cells, elevating ROS levels and triggering mitochondrial apoptosis, ultimately inducing cell death.

**Fig. 9:**
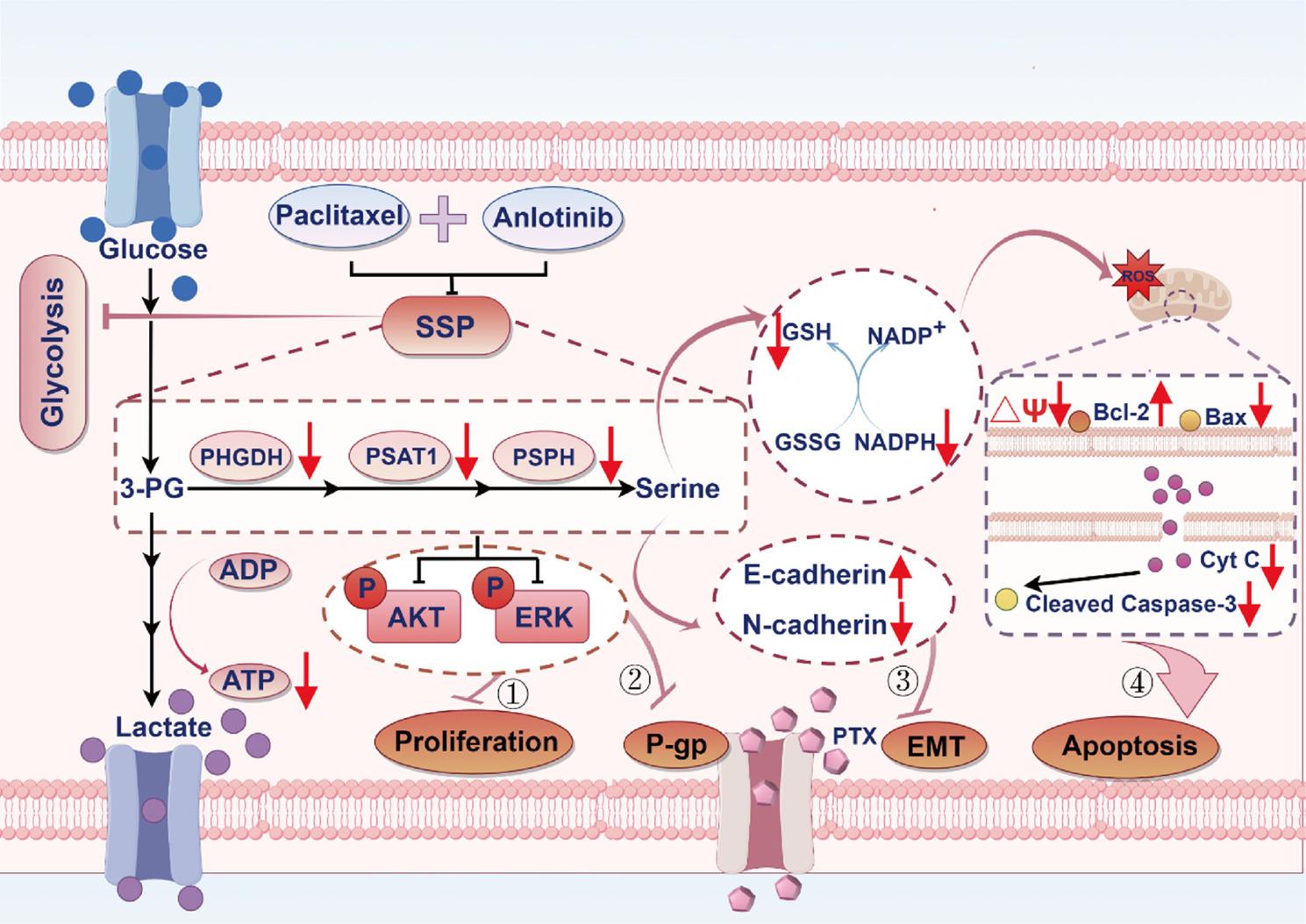
Graphical representation of SSP-driven PTX resistance pathways and potential intervention strategies. The combination of anlotinib and PTX effectively suppresses the SSP activity in A549/PTX cells. This suppression results in the following effects: (1) disruption of AKT/ERK proliferation signaling pathway transmission;(2) inhibition of P-gp expression and its efflux function; (3) blockade of the EMT process; and (4) activation of the mitochondrial apoptosis pathway, thereby inducing cell apoptosis. Furthermore, the inhibition of SSP activity also exerts a certain degree of suppression on the glycolytic activity of A549/PTX cells (Created by figdraw.com)

In conclusion, our study demonstrates that the SSP pathway mediates PTX resistance in NSCLC cells. As illustrated in Fig. 9, We show that combining anlotinib with PTX overcomes PTX resistance in A549/PTX cells through SSP inhibition. This reversal can be achieved through four distinct mechanisms: blocking the AKT/ERK proliferation signaling pathway and reducing glycolysis, thereby inhibiting cell proliferation; downregulating the expression of P-gp and its efflux function, thus preventing drug efflux; suppressing the EMT process, weakening cell migration and invasion capabilities and activating the mitochondrial apoptosis pathway to induce cell apoptosis. Collectively, these findings reveal a novel dual-drug combination strategy for overcoming PTX resistance in NSCLC, potentially providing a significant breakthrough in addressing this therapeutic challenge.

## Supplementary Information

Supplementary Material 1.

## Data Availability

All data supporting the findings of this study are available from the corresponding author upon reasonable request.
