# Supplementary material for: Targeting serine synthesis pathway to reverse paclitaxel resistance in NSCLC with combination of paclitaxel and anlotinib
Source: J Exp Clin Cancer Res. 2026 Jan 5;45:30. doi: 10.1186/s13046-025-03627-w (PMC12871031; doi:10.1186/s13046-025-03627-w)
Supplement: Supplementary file 1 — Supplementary Material 1. [file 13046_2025_3627_MOESM1_ESM.docx]

**Tittle page**

**Targeting serine synthesis pathway to reverse paclitaxel resistance in NSCLC with combination of paclitaxel and anlotinib**

**Mengting Yu^1^, Yanyun Hong^1,^, Qingshan Pan^1^, Pengwu Zheng^1^, Yingxing He^2^, Wufu Zhu^1,^*, Shan Xu^1,^* ,Qiaoli Lv^2^***

^1^Jiangxi Provincial Key Laboratory of Drug Design and Evaluation, School of Pharmacy, Jiangxi Science & Technology Normal University, Nanchang 330013, Jiangxi, China

^2^Jiangxi Key Laboratory of Oncology (2024SSY06041), JXHC Key Laboratory of Tumour Metastasis, Jiangxi Cancer Hospital & Institute, the Second Affiliated Hospital of Nanchang Medical College, Nanchang 330029, Jiangxi, China.

* Correspondence author Tel :+86 18827918863 (Shan Xu).

Correspondence E-mail addresses:

^2^Qiaoli Lv: lvqiaoli2008@126.com

^1^Shan Xu: shanxu9891@126.com

^1^Wufu Zhu: zhuwufu-1122@163.com.

**Supporting Information**

**Targeting serine synthesis pathway to reverse paclitaxel resistance in NSCLC with combination of paclitaxel and anlotinib**

**Materials and methods**

**Cell culture**

The human NSCLC cell line A549 (PTX-sensitive) was obtained from Jiangxi Cancer Hospital, while its PTX-resistant cells A549/PTX was purchased from Procell Life Science & Technology Co., Ltd (Wuhan, China). The A549 cells were cultured in DMEM medium, while the A549/PTX cells were grown in Ham's F-12K medium. Both media were enriched with 10 % FBS and 1 % penicillin/streptomycin, and the cultures were maintained at 37°C in a humidified incubator with 5% CO_2._

**Cell viability assay**

Cells were seeded in 96-well plates and treated with paclitaxel, anlotinib, their combination, or NCT-503 for 24-72 h. After treatment, each well received 20 μL of MTT solution (5 mg/mL). Following 4 h incubation, the resulting formazan deposits were solubilized with DMSO, and the absorbance was read at 490 nm using a Thermo microplate reader (Thermo, USA).

The interaction between paclitaxel and anlotinib was quantified through the calculation of the combination index (CI) using CompuSyn software (ComboSyn Inc., Paramus, NJ, USA), based on the Chou-Talalay method. In this context, CI values less than 1 suggest a synergistic relationship, a value of exactly 1 indicates an additive effect, and values greater than 1 point to an antagonistic interaction. A smaller CI indicates a stronger synergistic effect between the two drugs.

**Colony formation assay**

Cells were cultured in 6-well plates and treated with paclitaxel and/or anlotinib, or NCT-503 alone. The medium was refreshed every 48 h. After 14 days of culture, the medium was carefully aspirated. Colonies were gently washed three times with PBS, fixed with 4% formaldehyde solution for 15 min at room temperature, and then stained with 0.1% crystal violet for 15 min. After thorough washing with distilled water to remove excess dye, colonies were quantified using ImageJ software.

**EdU incorporation assay**

Cells were plated into 24-well plates and exposed to paclitaxel, anlotinib, a combination of paclitaxel and anlotinib, or NCT-503 for a duration of 72 h. The proliferation assay was conducted using the BeyoClick™ EdU-488 kit from Beyotime. Fluorescent microscopy (Olympus, Japan) was employed to capture images of EdU-positive cells, which were then quantified with ImageJ software.

**Rh123 accumulation assay**

A549 and A549/PTX cells were cultivated in 6-well plates and and treated them with specified drug concentrations for 72 h. The cells were then incubated with 1 μM Rh123 at 37°C for 30 min. Using a fluorescence microscope (Olympus, Japan), we captured fluorescence images and analyzed them with ImageJ software. Additionally, a flow cytometry assay was performed to evaluate the impact of the drugs on the intracellular accumulation of Rh123, and the results were examined with FlowJo software.

**Flow cytometric analysis**

A549/PTX cells were exposed to the designated drug concentrations for a duration of 72 h. For apoptosis analysis, the cells were incubated with Annexin V-FITC and propidium iodide in darkness for 15 min before being analyzed via flow cytometry (BD Accuri™ C6, USA). For cell cycle analysis, the cells were fixed in 70% cold ethanol at 4°C for 24 h, then washed twice with PBS and stained in darkness prior to flow cytometric analysis using the same instrument. The resulting data were processed with FlowJo software

**Wound-healing migration assay**

Cells were initially plated in 6-well plates and allowed to grow until reaching approximately 90% coverage. To inflict a straight-line scratch, a clean 200 μL pipette tip was gently dragged across the monolayer. After rinsing twice with PBS to eliminate any floating debris, the cells were exposed to the designated drug doses. Following a 48-hour incubation period, the migrated cells were observed with a fluorescence microscope (Olympus, Japan), and the extent of wound healing was measured using ImageJ software.

**Transwell assay**

The chamber surfaces were either coated with Matrigel (diluted 1:10, LYNJUNE) for the invasion assay or left without coating for the migration assay. After exposing cells to specified drug concentrations for 48 h , we trypsinized and counted them, then inoculated equal numbers of viable cells from each group, suspended in 200 μL of serum-free medium, into the upper Transwell chamber, with the lower chamber containing 500 μL of medium supplemented with 10% FBS. After a 48-hour incubation period, the cells that successfully moved through the membrane were fixed using 4 % formaldehyde for 15 min at room temperature, and then stained with 0.1% crystal violet for 15 min. Wash away the excess dye with pure water and gently wipe the chamber edge areas with a cotton swab.Images were then taken with an Olympus fluorescence microscope from Japan.

**Calcein-AM/PI staining**

A549/PTX cells were cultured in 24-well plates and exposed to designated drug concentrations for 48 h. The cells were first stained with Calcein AM (2 μM) for 20 min, followed by propidium iodide (PI, 5 μM) for 5 min. After washing with PBS to remove residual staining reagents, the cells were observed under a fluorescence microscope (Olympus, Japan).

**Enzyme activity assay**

Using the PHGDH Activity Assay Kit (ab273328), enzyme activity is determined based on the following principle: PHGDH catalyzes the conversion of 3-phosphoglycerate and NAD to 3-phosphohydroxypyruvate and NADH, respectively. The generated NADH then reacts with a colorimetric probe, producing a stable increase in absorbance at 450 nm. The assay is conducted at 37°C, and absorbance is measured in kinetic mode at one-minute intervals over a period of 30 minutes. Sample preparation involves protein extraction, centrifugation, and ammonium sulfate precipitation to remove interfering substances. Enzyme activity (U/mL) is subsequently calculated using a NADH standard curve.

**
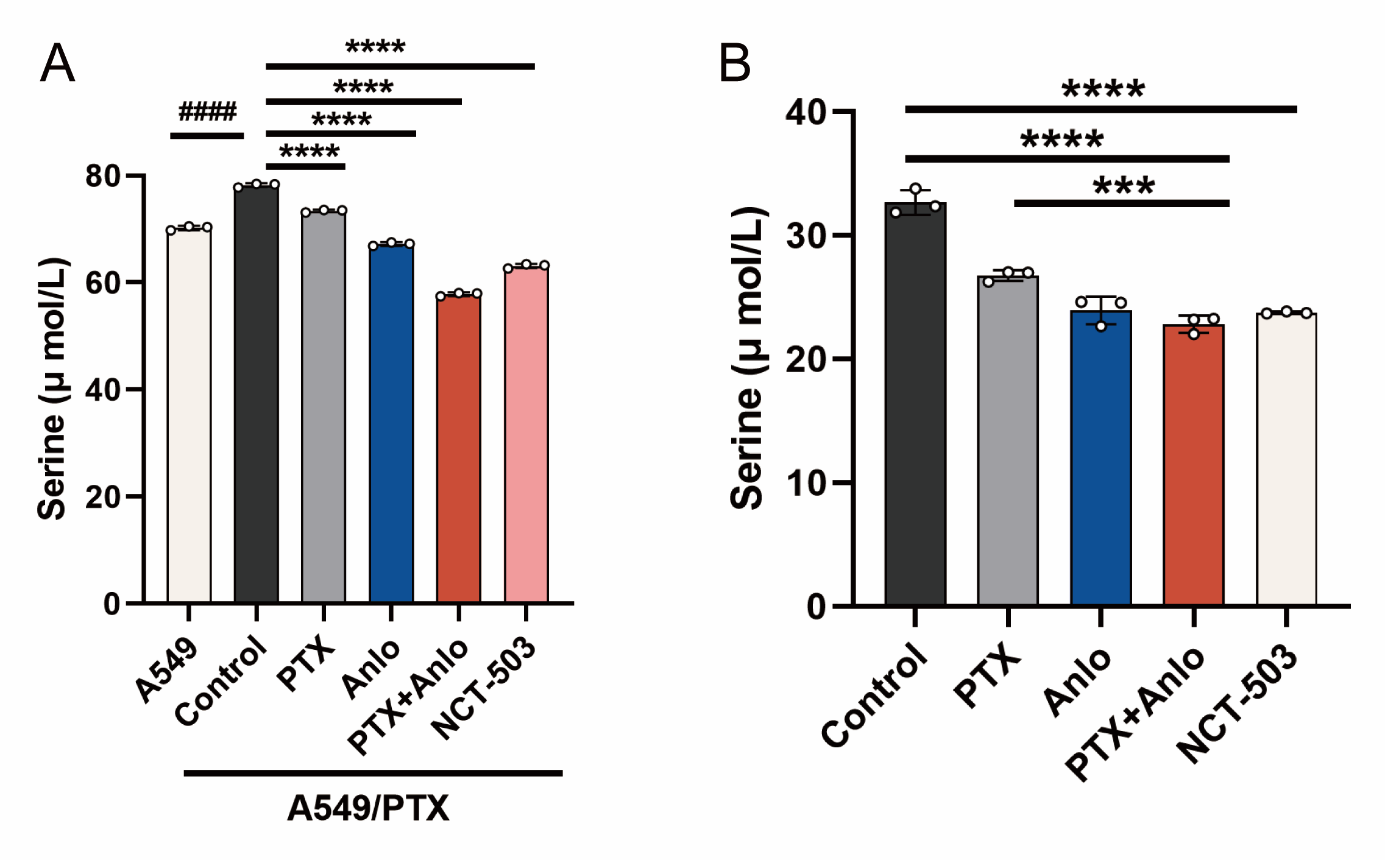
**

**Fig. S1** **The combination treatment with anlotinib and paclitaxel significantly reduced serine levels in both A549/PTX cells and tumor tissues from A549/PTX xenograft-bearing nude mice (n = 3)**.


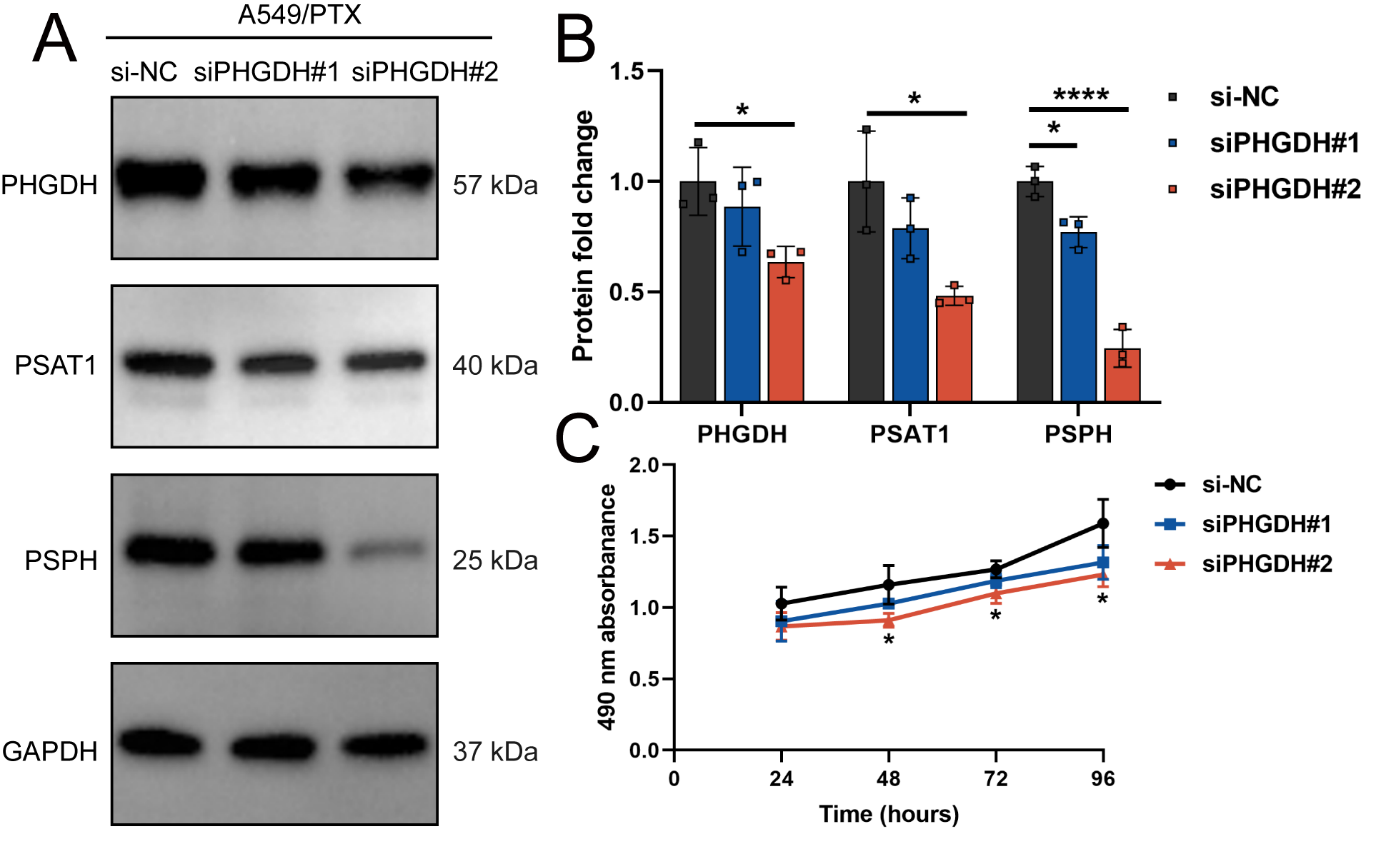


**Fig. S2** **Suppression of PHGDH by siRNA knockdown inhibits the SSP of A549/PTX cells**. **A,B**. The expression of SSP proteins in A549/PTX cells after transfection with PHGDH-specific siRNAs was measured by western blotting (n = 3). **C**. MTS assay was performed to assess cell viability (n = 3).


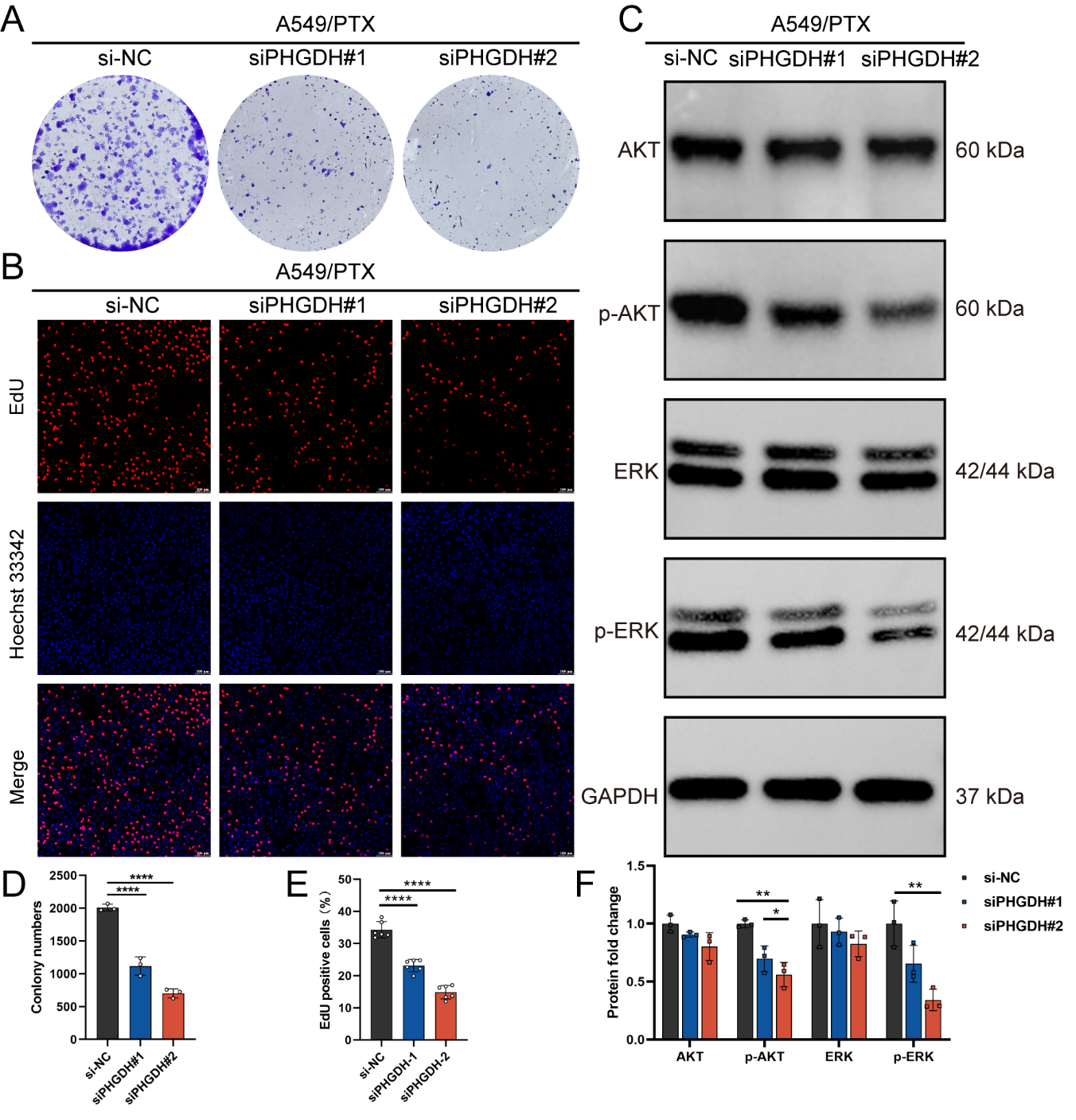


**Fig. S3 Transfection of A549/PTX cells with siPHGDH impaired cell proliferation by disrupting the AKT/ERK signaling pathway**. **A,D**. Colony formation assays (n = 3). **B,E**. EdU assays (scale bar: 100 μm; n = 3). **C,F**. Western blotting analysis of AKT/ERK signaling pathway proteins (n = 3).


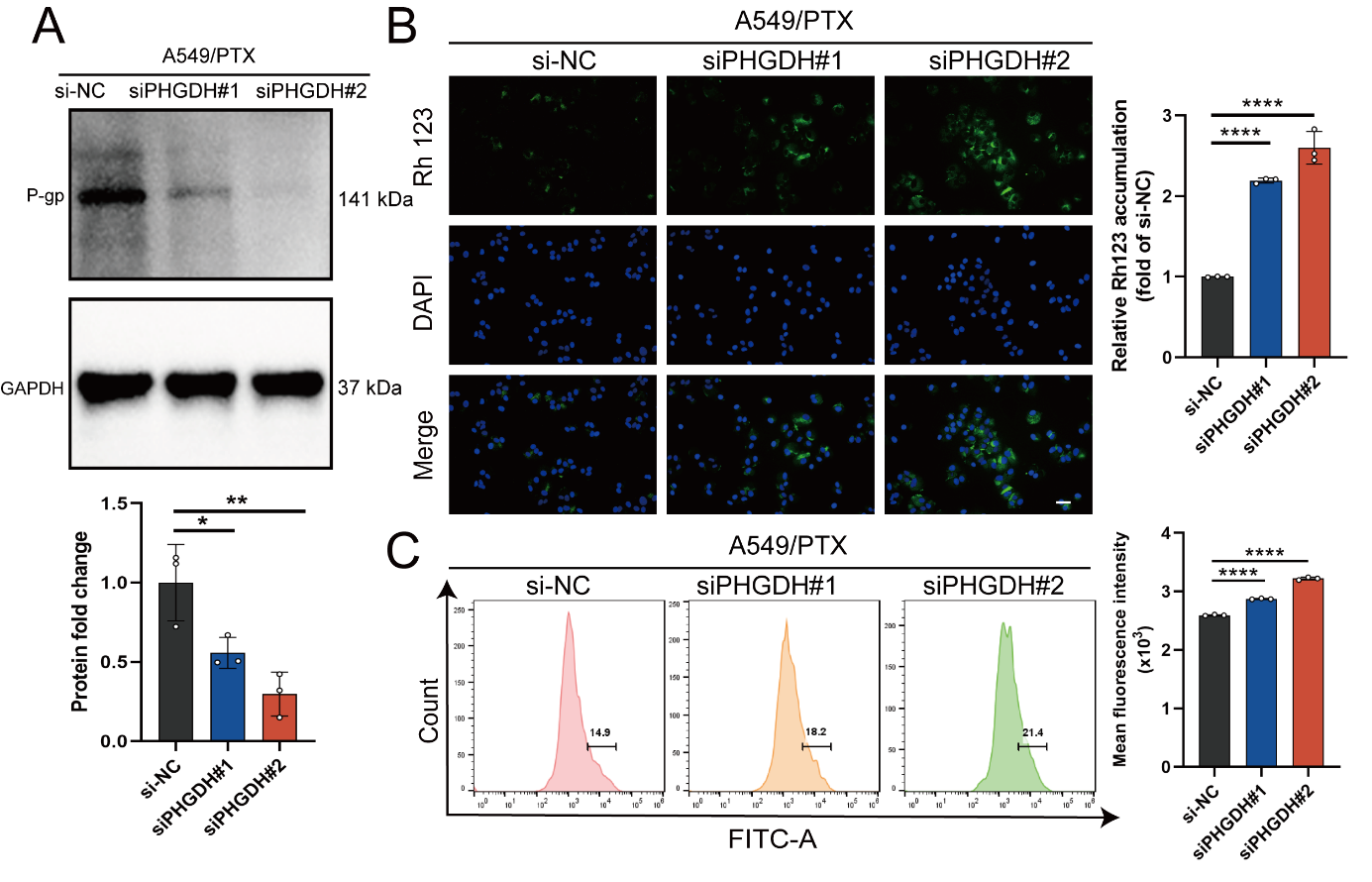


**Fig. S4 Transfection of A549/PTX cells with siPHGDH reduces P-gp expression and transport function in A549/PTX cells.** **A**. Western blotting analysis of P-gp proteins (n = 3). **B**. Fluorescence microscopy (Scale bar: 100 μm; n = 3 ) and **C**. flow cytometry to detect Rh123 accumulation in A549/PTX cells (n = 3).

**
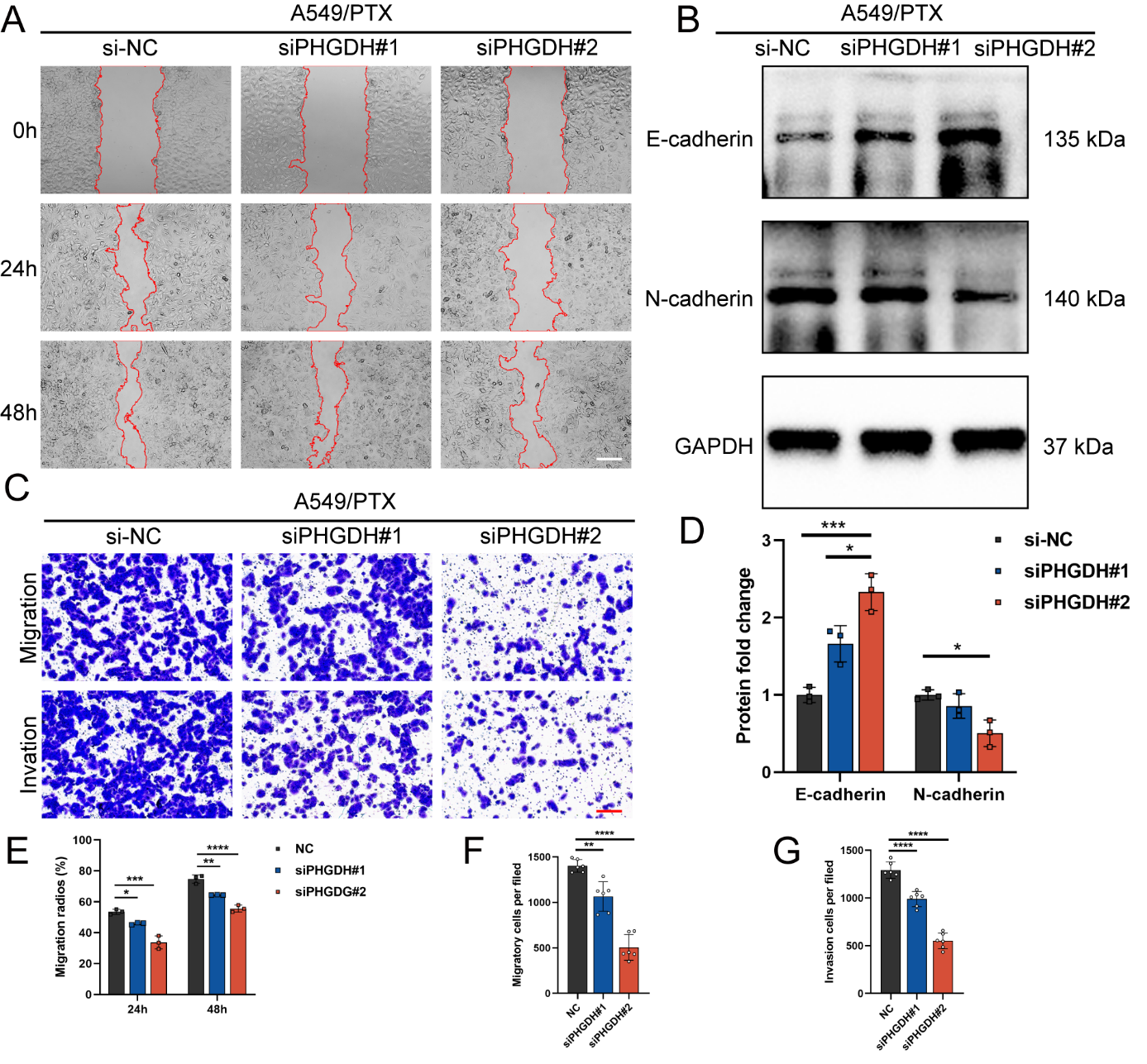
**

**Fig. S5 Transfection of A549/PTX cells with siPHGDH reverses the EMT process in A549/PTX cells.** **A,E**. Wound-healing migration assay (scale bar: 200 μm; n = 3). **B,D**. Western blotting of EMT markers (n = 3). **C,F,G**. Transwell assay (n = 6).


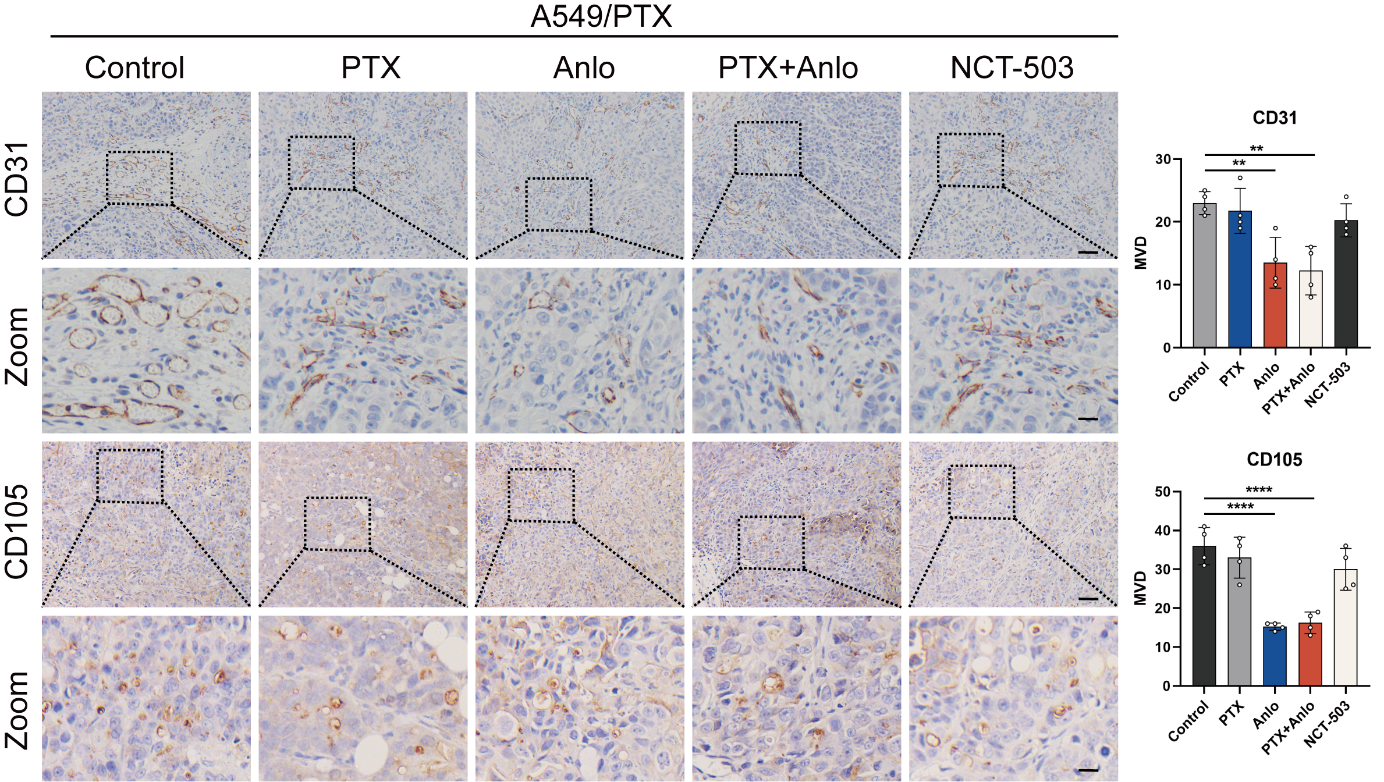


**Fig. S6 Immunohistochemical detection of microvessel density after the combined treatment with anlotinib and paclitaxel** **(scale bar: 200 μm; Zoom scale bar: 20 μm , n = 4).**
